# Transcriptome analysis of the responses of *Staphylococcus aureus *to antimicrobial peptides and characterization of the roles of *vraDE *and *vraSR *in antimicrobial resistance

**DOI:** 10.1186/1471-2164-10-429

**Published:** 2009-09-14

**Authors:** Milla Pietiäinen, Patrice François, Hanne-Leena Hyyryläinen, Manuela Tangomo, Vera Sass, Hans-Georg Sahl, Jacques Schrenzel, Vesa P Kontinen

**Affiliations:** 1 Antimicrobial Resistance Unit, Department of Infectious Disease Surveillance and Control, National Institute for Health and Welfare (THL), PL 30, 00271 Helsinki, Finland; 2 Genomic Research Laboratory, Service of Infectious Diseases, University Hospitals of Geneva, University of Geneva, 1211 Geneva-14, Switzerland; 3 Institute for Medical Microbiology, Immunology and Parasitology, Pharmaceutical Microbiology Unit, University of Bonn, Bonn, Germany

## Abstract

**Background:**

Understanding how pathogens respond to antimicrobial peptides, and how this compares to currently available antibiotics, is crucial for optimizing antimicrobial therapy. *Staphylococcus aureus *has several known resistance mechanisms against human cationic antimicrobial peptides (CAMPs). Gene expression changes in *S. aureus *strain Newman exposed to linear CAMPs were analyzed by DNA microarray. Three antimicrobial peptides were used in the analysis, two are derived from frog, temporin L and dermaseptin K4-S4(1-16), and the ovispirin-1 is obtained from sheep.

**Results:**

The peptides induced the VraSR cell-wall regulon and several other genes that are also up-regulated in cells treated with vancomycin and other cell wall-active antibiotics. In addition to this similarity, three genes/operons were particularly strongly induced by the peptides: *vraDE*, SA0205 and SAS016, encoding an ABC transporter, a putative membrane-bound lysostaphin-like peptidase and a small functionally unknown protein, respectively. Ovispirin-1 and dermaseptin K4-S4(1-16), which disrupt lipid bilayers by the carpet mechanism, appeared to be strong inducers of the *vraDE *operon. We show that high level induction by ovispirin-1 is dependent on the amide modification of the peptide C-terminus. This suggests that the amide group has a crucial role in the activation of the Aps (GraRS) sensory system, the regulator of *vraDE*. In contrast, temporin L, which disrupts lipid bilayers by forming pores, revealed a weaker inducer of *vraDE *despite the C-terminal amide modification. Sensitivity testing with CAMPs and other antimicrobials suggested that VraDE is a transporter dedicated to resist bacitracin. We also showed that SA0205 belongs to the VraSR regulon. Furthermore, VraSR was shown to be important for resistance against a wide range of cell wall-active antibiotics and other antimicrobial agents including the amide-modified ovispirin-1, bacitracin, teicoplanin, cefotaxime and 10 other β-lactam antibiotics, chlorpromazine, thioridazine and EGTA.

**Conclusion:**

Defense against different CAMPs involves not only general signaling pathways but also CAMP-specific ones. These results suggest that CAMPs or a mixture of CAMPs could constitute a potential additive to standard antibiotic treatment.

## Background

The ubiquitous presence of cationic antimicrobial peptides (CAMPs) in virtually all types of cells and organisms ranging from bacterial cells to humans suggests that CAMPs have important conserved roles as defense weapons. CAMPs contribute to the host defense of microbial invasion on epithelial surfaces by killing engulfed micro-organisms in phagocytic cells or modulating inflammatory responses in infections [1-3]. These ancient weapons of host defense are typically amphipathic peptides with a net positive charge at physiological pH and they share well-defined α-helical or β-sheet secondary structures. CAMPs are able to integrate into cell membranes, form membrane-spanning pores and thereby cause lethal cell damage. The positive charge is important for the initial binding of CAMPs to target membranes. There are differences in the way CAMPs interact with membranes and accordingly three different models have been used to define their mode of actions in model membrane systems [4]. In the *barrel-stave mechanism*, peptides integrate into the membrane and form membrane-spanning pores [5]. In the *toroidal-pore mechanism*, CAMPs form membrane-spanning pores together with intercalated lipids [5]. And in the *carpet mechanism*, peptides accumulate on the membrane surface in a carpet-like manner and at a threshold density so that they dissolve the membrane without forming transmembrane channels [6]. However, membrane damage is not the only mechanism whereby CAMPs cause cell death. They may also affect functions of several other cell components and act as metabolic inhibitors of cellular processes including biosynthesis of the cell wall, nucleic-acids and proteins [1,3]. In these cases, the cell death can be the result of multiple inhibitory effects.

Bacteria have evolved mechanisms to combat the harmful effects of CAMPs [3]. On the other hand, the antimicrobial peptide repertoire in host cells may have co-evolved with the evolution of microbial resistance mechanisms. This is exemplified by the skin of the frog *Rana temporaria *in which some temporins act in a synergistic manner to overcome the resistance of Gram-negative bacteria imposed by the lipopolysaccharide [7]. The high resistance of *Staphylococcus aureus *to CAMPs produced by human cells may contribute to epithelial colonization and resistance to destruction by neutrophils. Several mechanisms conferring increased resistance to CAMPs have been identified. The modulation of the density of negative charge in the cell wall by D-alanylation of teichoic acids (Dlt system) or on the outer surface of the cell membrane by L-lysinylation of phosphatidylglycerol (MprF) has been shown to contribute to CAMP resistance in *S. aureus *[8,9] as well as in several other bacteria [10-13]. In Gram-negative bacteria, LPS modifications modulate cell envelope charge and polymyxin susceptibility [14-18]. Many bacteria species produce proteases which cleave antimicrobial peptides, particularly linear ones [3]. *S. aureus *aureolysin and V8 as well as proteases secreted by *Bacillus anthracis *are examples of proteases capable of cleaving the human cathelicidin LL-37 [19,20]. The CAMP resistance mechanisms also include CAMP-binding proteins and CAMP efflux pumps [21-23].

Bacterial sensory systems are capable of recognizing cationic antimicrobial peptides and to respond to their presence by up-regulation of general stress systems as well as more specific CAMP resistance mechanisms. In *Salmonella typhimurium *binding of CAMPs to the PhoQ two-component sensor kinase activates the signal transduction cascade from the sensor to the PhoQ response regulator, resulting consequently in the induction of the PhoPQ-regulated promoters [24]. A complex stress response was observed when *Bacillus subtilis *was exposed to CAMPs, including activation of the SigW and SigM extracytoplasmic sigma factors and the YxdJK and LiaRS two-component systems [25]. In this study, our purpose was to characterize the stress response of *S. aureus *treated with cationic antimicrobial peptides by using whole-genome oligoarrays. The effects of three different α-helical CAMPs on the transcriptome of the *S. aureus *Newman strain were analyzed: temporin L, ovispirin-1 and dermaseptin K4-S4(1-16). These peptides are synthesized as preproproteins (precursors) and the mature microbicidal peptides are formed after proteolytic cleavage of the pre- (signal peptide) and pro-regions of the precursors [26,27]. Ovispirin-1 is a derivative of the cathelicidin SMAP-29 found in sheep [28]. The other two peptides, dermaseptin K4-S4(1-16), which is a truncated derivative of the dermaseptin S4, and temporin L, are both expressed in amphibian skin [29,30]. Temporin L belongs to pore-forming peptides (barrel-stave or toroidal pore mechanism) [30,31] and ovispirin-1 and dermaseptin disrupt lipid bilayers by the carpet mechanism [1]. We used these well-characterized peptides of animal origin as models of cationic antimicrobial peptides and expected that studying their interactions with *S. aureus *would give information that was more generally applicable also to human CAMPs. We aimed to identify the CAMP stimulons and to determine whether the differentially expressed genes play a role in CAMP resistance. Furthermore, we were interested to find out whether there are strong peptide-specific responses or a more general stress response triggered by CAMPs exposition, to explore the mode of action of CAMPs and to improve our understanding of the resistance mechanisms against CAMPs.

## Results

### The VraSR cell wall regulon, vraDE, SA0205 and SAS016 are strongly induced by cationic antimicrobial peptides

Exponentially growing *S. aureus *cells were treated with C-terminally amidated temporin L (temporin L-NH_2_), ovispirin-1 (ovispirin-1-NH_2_) or dermaseptin K4-S4(1-16) (dermaseptin K4-S4(1-16)-NH_2_) at sublethal concentrations (see Methods) which slightly inhibited growth but did not stop it. Gene expression changes in the CAMP-treated cells as compared to non-treated cells were analyzed by using whole-genome oligoarrays.

All three peptides upregulated a large number of genes (63-247) and most of them were induced by more than one peptide. Table 1 shows a set of the genes induced by at least one of the peptides at least 3-fold and represents a list of "marker" genes for the CAMP stimulon. The complete list of the induced genes (higher than 2-fold induction) is shown in additional file 1. The microarray data sets have been deposited in the GEO database (GPL7137 and GSE15800 for the complete microarray dataset) [32].

**Table 1:** Genes induced by cationic antimicrobial peptides.

**Gene ID**	Gene name*	Induction with**			Known regulation***	**Protein/Similarity**
				
		**T**	**O**	**D**		
SA0011		**3.5**	**2.1**			**Similar to homoserine-o-acethyltransferase**
SA0122	***butA***	**3.2**	**2.1**		**VCM**	**Acetoin reductase**
SA0205		**17.2**	**11.2**	**6.2**	**VCM**	**Similar to lysostaphin precursor**
SA0344	***metE***	**4.5**	**2.3**		**VCM**	**5-methyltetrahydropteroyltriglutamate-homocysteine methyltransferase**
SA0428		**3.6**	**2.4**	**2.3**		**Hypothetical protein**
SA0430	***gltB***	**4.4**	**2.5**			**Glutamate synthase large subunit**
SA0480	***ctsR***	**4.2**	**4.2**			**Transcription repressor of class III stress genes homologue**
SA0513		**3.3**	**2.2**			**Conserved hypothetical protein**
SA0591		**3.0**	**3.3**	**2.1**	**VCM**	**Hypothetical protein**
SA0677		**3.6**	**2.1**		**VCM**	**Similar to choline transport ATP-binding protein**
SA0781		**3.1**	**2.1**			**Similar to 2-nitropropane dioxygenase**
SA0817		**5.0**	**2.7**			**Similar to NADH-dependent flavin oxidoreductase**
SA0825	***spsA***	**2.6**	**3.5**	**2.0**	**VraSR, VCM**	**Type-1 signal peptidase**
SA0835	***clpB***	**3.9**	**4.1**		**VCM**	**ClpB chaperone homologue**
SA0845	***oppB***	**4.3**	**2.0**		**VCM**	**Oligopeptide transport system permease protein**
SA0903		**2.1**	**4.1**	**2.6**		**Conserved hypothetical protein **
SA1164	***dhoM***	**5.2**	**2.8**		**VCM**	**Homoserine dehydrogenase**
SA1170	***katA***	**4.3**	**2.6**			**Catalase**
SA1216		**3.8**	**2.4**			**Similar to oligoendopeptidase**
SA1219			**5.4**	**4.2**		**Similar to phosphate ABC transporter**
SA1227	***dapA***	**8.3**	**9.6**		**VCM**	**Dihydrodipicolinate synthase**
SA1254		**3.1**	**3.5**		**VraSR, VCM**	**Hypothetical protein**
SA1476		**4.2**	**5.3**	**3.3**	**VraSR, VCM**	**Hypothetical protein**
SA1517	***citC***	**4.9**	**5.8**	**3.1**	**VCM**	**Isocitrate dehydrogenase**
SA1545	***serA***	**4.6**	**2.5**		**VCM**	**Similar to soluble hydrogenase 42 kD subunit**
SA1549	***htrA***	**2.9**	**3.0**	**2.0**	**VraSR, VCM**	**Similar to serine proteinase Do, heat-shock protein HtrA**
SA1599		**3.2**	**2.4**			**Similar to transaldolase**
SA1655	***ecsA***	**3.0**	**3.0**			**ABC transporter EcsA homologue**
SA1659	***prsA***	**3.2**	**4.5**	**2.9**	**VraSR, VCM**	**Peptidyl-prolyl *cis*/*trans *isomerase homologue**
SA1701	***vraS***	**3.9**	**5.3**	**2.5**	**VraSR, VCM**	**Two-component sensor histidine kinase**
SA1820		**3.6**	**3.0**	**2.2**		**Similar to bacteriophage terminase small subunit**
SA1836	***groEL***	**3.8**	**4.3**	**2.2**		**GroEL protein**
SA1862	***leuA***	**13.5**	**3.3**	**2.1**		**2-isopropylmalate synthase**
SA1990		**3.4**	**2.6**	**2.2**		**Conserved hypothetical protein**
SA2113		**2.2**	**3.7**	**2.2**	**VraSR, VCM**	**Hypothetical protein**
SA2221		**2.4**	**3.2**		**VraSR, VCM**	**Hypothetical protein**
SA2304	***fbp***	**4.8**	**2.4**		**VCM**	**Fructose-bisphosphatase**
SA2324		**4.9**	**2.6**	**2.0**		**Similar to thioredoxin**
SA2343		**4.3**	**4.3**	**2.7**	**VraSR, VCM**	**Hypothetical protein**
SA2397		**3.5**	**2.3**		**VCM**	**Similar to pyridoxal-phosphate dependent aminotransferase**
SA2467	***hisH***	**40.1**	**4.3**		**VCM**	**Amidotransferase HisH**
SA2492	***vraD***	**8.2**	**32.4**	**17.2**	**VCM**	**Similar to ABC transporter**
SAS016		**5.0**	**7.4**	**5.4**	**VCM**	**Hypothetical protein**

*: Only the most strongly upregulated genes, the marker genes of the CAMP stimulon, are shown in this table. The complete list of genes induced at least 2-fold by several CAMPs is shown in additional file 1. In the case of operons, only the first gene or the most strongly induced gene is shown;

**: T, temporin L-NH_2_; O, ovispirin-1-NH_2_; D, dermaseptin K4-S4(1-16)-NH_2_;

***: VCM, vancomycin inducible; VraSR, belongs to the VraSR regulon.

CAMPs induced the expression of the VraSR two-component system and consequently almost the whole VraSR regulon [33] was upregulated. In addition to the three antimicrobial peptides of animal origin, we also recently found that the human cathelicidin LL-37 induces the VraSR regulon (data not shown). Among the most strongly induced genes were *vraDE*, SA0205 and SAS016, which encode an ABC-type transporter similar to a putative bacitracin efflux pump [34,35], a lysostaphin-like cell-wall peptidase and a functionally-unknown peptide of 55 amino acids, respectively. The antimicrobial peptide treatment caused stress that induced general stress genes such as the *ctsR*-*clpC *operon, *groELS *and *dnaJK*. The synthesis of cell components involved in combating oxidative stress, catalase (SA1170), a putative thioredoxin (SA2324) and thioredoxin reductases (*trxB*), were also among the induced genes. Furthermore, a feature of the transcriptome was upregulation of several amino acid-biosynthesis operons (*dap, his*, *leu*, and *thr *operons).

### Genes involved in anaerobic energy metabolism or encoding virulence factors were down-regulated in CAMP-treated cells

There were also down-regulated genes in the transcriptomes. Temporin L-NH_2_, ovispirin-1-NH_2 _and dermaseptin K4-S4(1-16)-NH_2 _decreased the expression of 219, 194 and 134 genes, respectively. Most of the genes were down-regulated by more than one peptide (additional file 2). Notably, all three peptides had a strong inhibitory effect on the expression of genes involved in energy metabolism under anaerobic conditions [36]. The genes encoding enzymes for nitrate respiration (*nar *and *nas *operons) and fermentation (*pflP*, *pflA*, *ictE*, and *adh1*) were strongly repressed. Another striking phenomenon is that antimicrobial peptides caused down-regulation of several virulence factors and their regulators (*saeRS *and *agr*). Among the down-regulated virulence factor genes were *hld*, *ssaA*, *sbi*, *hlgA*, *ssl11 *(*set15*), *clfA*, *clfB *and *spa*. The negative effect was particularly strong on the expression of *hld*, *ssl11*, *clfA *and *clfB *in cells treated with ovispirin-1-NH_2_.

### qRT-PCR measurements of the differentially expressed genes

We used qRT-PCR to verify some of the most interesting gene expression responses. The most strongly upregulated genes of the transcriptome,*vraD *(SA2492), SA0205 and SAS016 were subjected to qRT-PCR. The activity of the VraSR regulon was determined with three genes, *vraS *(SA1701), *prsA *(SA1659) and SA1477. The DNA microarray data suggested that an operon encoding a putative PstB-like phosphate uptake system (SA1217-SA1221) was induced by ovispirin-1-NH_2 _and dermaseptin K4-S4(1-16)-NH_2 _but not by temporin L-NH_2 _(Table 1 and additional file 1). This induction pattern suggests that the peptide-induced expression from the promoter of the SA1217-SA1221 operon may depend on the mode of action of the antimicrobial peptides and we therefore were interested in confirming the peptide-specific induction pattern by qRT-PCR. We determined the expression level of SA1220. We also determined two further genes, *vraF *(SA0616) and *dltA *(SA0793), which were induced about 2-fold in the microarrays only by ovispirin-1-NH_2_. The ortholog of *vraF *in *B. subtilis *is *bceA*, which encodes the ATPase component of an ABC transporter. Since *bceA *is strongly induced by bacitracin [34] and moderately by linear cationic antimicrobial peptides [25], and since it is also important for bacitracin resistance, we were interested in determining whether *vraF *is induced in the CAMP-treated cells of *S. aureus*. Furthermore, *vraDE*, *vraFG *and the *dlt *operon are all regulated by the Aps (or GraRS) sensory system [37]. The *dlt *operon encodes proteins which modify wall teichoic acids and lipoteichoic acids with D-alanine and thereby modulate the cell wall charge [9]. The density of negative charge in the wall affects the sensitivity of the bacteria to CAMPs.

**Figure 1:**
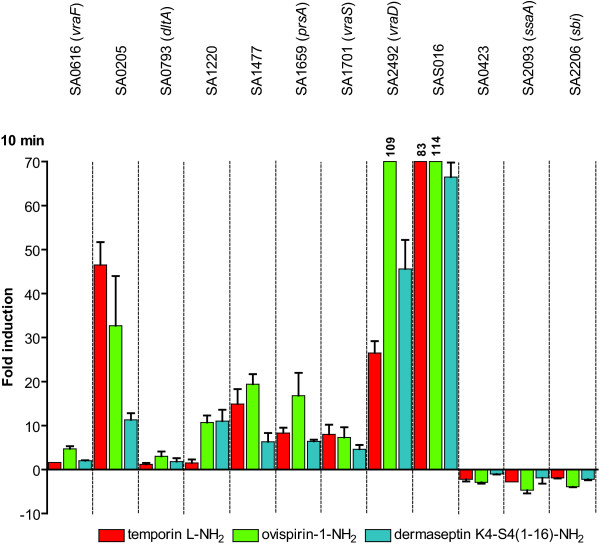
**Induction or repression of genes in cells treated with cationic antimicrobial peptides as determined by qRT-PCR**. The measurements of a set of differentially expressed genes were performed after 10 min treatments. The standard deviations of SAS016 are 19 (temporin L-NH_2_) and 53 (ovispirin-1-NH_2_). The standard deviation of *vraD *(ovispirin-1-NH_2_) is 6.

The gene expression changes of the induced genes were clearly higher in the qRT-PCR measurements than in the DNA microarray, a previously described characteristic [38,39], but overall consistency was found in the results. The SAS016, *vraD*, SA0205, SA1477 and *prsA *genes exhibited the strongest gene induction responses; over 100-fold induction of SAS016 and *vraD *was observed with ovispirin-1-NH_2 _(Figure 1). Most of the genes responded more strongly to ovispirin-1-NH_2 _than temporin-L-NH_2_. This was particularly clear in the induction of *vraD *and SA1220, but also *vraF*, *dltA*, *prsA*, SAS016 and SA1477 responded in this manner. SA0205 was the only gene which was expressed at higher levels in temporin-L-NH_2_-treated cells as compared to ovispirin-1-NH_2_-treated cells. The gene expression responses to dermaseptin K4-S4(1-16)-NH_2 _were in most cases similar to those of ovispirin-1-NH_2_.

The negative effect of CAMPs on the expression of *ssaA*, *sbi *and SA0423 was verified by qRT-PCR. Consistently with the microarray results, these genes were down-regulated in cells treated with the peptides (Figure 1). However, the decrease in expression was clearly lower in the qRT-PCR measurements.

### The amide at the C-terminus of ovispirin-1 is crucial for the high level induction of vraDE

The three peptides used in the DNA microarray analysis were modified with C-terminal amide groups. We studied whether the amide affects gene induction by comparing the induction levels of four key genes in cells treated with amide-modified or non-modified ovispirin-1 or amide-modified temporin L. The determined genes were *groEL*, a general stress gene, *prsA*, the activity of which reflects the activity of the VraSR two-component system and the severity of the stress in the cell wall [33], and SA0205 and *vraD*, which were strongly induced by CAMPs. We also determined how other types of antimicrobial agents induce these genes. The antimicrobial agents were vancomycin and teicoplanin, which inhibit cell wall biosynthesis by interacting with the lipid II peptidoglycan precursor [40], bacitracin, an antimicrobial agent which interferes with the dephosphorylation of the peptidoglycan precursor [41], and daptomycin, a lipopeptide antibiotic with a specific mode of action on the cell membrane [42]. Furthermore, a pentaglycin peptide, A(D-Glu)K(D-Ala)GGGGGA(D-Glu)K(D-Ala), which mimics the peptide cross-link of *S. aureus *peptidoglycan, was tested to see whether a native cell wall peptide induces the expression of the four genes. This peptide was not antimicrobial at the concentration used, 20 μg/ml.

The results revealed that the C-terminal amide strongly influenced the induction of *vraD *by ovispirin-1. In the absence of the amide, *vraD *was only induced 3.7-fold (Table 2). In contrast, when ovispirin-1 was modified with the amide, *vraD *was induced 136-fold. The MIC values of both of the ovispirin-1 peptides for the *S. aureus *Newman strain were 20 μg/ml (Table 3), indicating that a difference in the stability of the peptides does not explain the large difference (about 35-fold) in the induction levels. The amide-modified peptide was also a better inducer of SA0205, *prsA *and *groEL*, but the difference was much smaller, only 2-4-fold. The induction pattern of the cell wall antibiotics vancomycin and teicoplanin was similar to that of ovispirin-1 without the C-terminal amide: the induction levels of SA0205 and *prsA *were clearly higher than those of *vraD *and *groEL*. The *vraD *expression was also strongly induced by bacitracin, almost 500-fold, whereas the induction levels of SA0205 and *prsA *were much lower, 30-50-fold. Thus the induction pattern was similar to the amide-modified ovispirin-1. In contrast, temporin L-NH_2_, despite the C-terminal amide and lower MIC than ovispirin-1-NH_2_, was a poor inducer of *vraD *(Table 2, Figure 1) and resembled in this respect the non-modified ovispirin-1 and the cell wall antibiotics. The pentaglycine peptide did not induce any of the four genes.

**Table 2:** Induction of *vraD*, SA0205, *prsA *and *groEL *in *S. aureus *cells treated with peptides or cell wall-active antibiotics.

Antimicrobial agent and concentration**	Fold induction*			
	
	**Gene**			
	
	***vraD***	**SA0205**	***prsA***	***groEL***
Ovispirin-1 100 μg/ml	3.7 (0.5)	5.1 (1.0)	7.2 (1.6)	2.5 (0.5)
Ovispirin-1-NH_2_ 100 μg/ml	136.7 (8.7)	20.0 (0.7)	30.7 (1.7)	3.8 (0.4)
Temporin-L-NH_2_ 2 μg/ml	4.6 (1.3)	15.1 (3.6)	6.4 (1.8)	4.0 (0.1)
Bacitracin 100 μg/ml	467.5 (15.3)	51.4 (0.8)	27.9 (4.5)	3.6 (0.1)
Vancomycin 5 μg/ml	6.6 (0.6)	25.5 (1.8)	32.7 (1.7)	1.9 (0.0)
Teicoplanin 2.5 μg/ml	2.5 (0.3)	35.7 (6.9)	32.1 (4.1)	3.0 (0.3)
Pentaglysin 20 μg/ml	1.0 (0.0)	1.3 (0.1)	1.3 (0.0)	1.1 (0.0)

*: The ratio of the expression level of the gene in *S. aureus *Newman treated for 10 minutes with the antimicrobial agent or peptide to that in untreated cells as determined by qRT-PCR. The induction ratios are means from three experiments. The standard deviations are in parenthesis;

**: The concentrations of the antimicrobials and peptides used in the analysis are 5 × MIC (see Table 3) with the exception of temporin-L-NH_2_, which was used at a subinhibitory concentration, and pentaglycine, which was not microbicidal at the 20 μg/ml concentration.

**Table 3:** Antimicrobial sensitivity of the Δ*vraDE *and Δ*vraSR *mutants and their parental strain *S. aureus *Newman.

**Antimicrobial agent**	**MIC (μg ml^-1^)**		
	
	**Δ*vraSR*/*S. aureus *Newman**	**Δ*vraDE*/*S. aureus *Newman**	***S. aureus *Newman**
Ovispirin-1	20	20	20
Ovispirin-1-NH_2_	10	20	20
Temporin L-NH_2_	6	6	6
Nisin	6.25	6.25	6.25
Bacitracin	5	2.5	20
Daptomycin	2.5	2.5	2.5
Teicoplanin	0.125	0.5	0.5
Vancomycin	1	1	1

### VraDE is an ABC transporter system dedicated to resist bacitracin, whereas VraSR two-component system affects the resistance against a wider spectrum of antimicrobial agents

Our experiments showed that the VraDE ABC transporter and the VraSR two-component system are two CAMP-inducible systems which could be important for bacterial cells in resisting the harmful effects of CAMPs and possibly other antimicrobial agents. In order to study this, we constructed *vraDE *and *vraSR *null mutants and cultivated the mutants and the parental *S. aureus *Newman strain in Mueller-Hinton broth in the presence of various concentrations of the antimicrobials (two-fold serial dilutions) and determined their MIC values (Table 3). The CAMPs used in the susceptibility tests were, ovispirin-1-NH_2_, ovispirin-1, temporin L-NH_2_, two lantibiotics nisin and Pep5, human cathelicidin LL-37, and hBD3 defensin (the latter three are not shown in Table 3). In addition, we determined MICs for vancomycin, teicoplanin, bacitracin and daptomycin.

The mutations were verified by PCR (see methods), but also the expression of the *vraD*, SA0205, *prsA *and *groEL *genes in the *vraDE *and *vraSR *null mutants exposed to vancomycin was determined by qRT-PCR. The Δ*vraDE *mutant did not express *vraD *mRNA, consistent with the mutation (Table 4). The SA0205, *prsA *and *groEL *genes were expressed at about the same level as in the wild-type, 3-60-fold induction (Tables 4). In the Δ*vraSR *mutant, SA0205 and *prsA *were expressed at a low level (3-fold induction) consistent with the VraSR defect and suggesting that not only *prsA *but also SA0205 belong to the VraSR regulon. Their induction at a low level in the absence of VraSR may indicate that the expression is also controlled by another sensory system.

**Table 4:** Induction of *vraD*, SA0205, *prsA *and *groEL *in *S. aureus *Δ*vraSR *and Δ*vraDE *mutants treated with vancomycin.

**Mutant**	Fold induction*			
	
	**Gene**			
	
	***vraD***	**SA0205**	***prsA***	***groEL***
Δ*vraSR*	7.9 (0.2)	2.5 (0.2)	3.2 (0.1)	2.1 (0.1)
Δ*vraDE*	not expressed	60.4 (2.1)	36.0 (0.7)	3.1 (0.4)
wild type	6.6 (0.6)	25.5 (1.8)	32.7 (1.7)	1.9 (0.0)

*: The ratio of the expression level of the gene in the mutant or the wild-type strain treated for 10 minutes with 5 μg/ml of vancomycin to that in untreated cells as determined by qRT-PCR. The induction ratios are means from three experiments. Standard deviations are in parenthesis.

The Δ*vraDE *mutant was clearly more sensitive than the wild-type strains to bacitracin (about 10-fold difference in MIC), but no other differences were observed in the antimicrobial sensitivities. This strongly suggests that VraDE is a bacitracin-specific detoxification pump. More differences were observed with the Δ*vraSR *mutant. It exhibited increased sensitivity to teicoplanin (4-fold difference), bacitracin (4-fold difference) and ovispirin-1-NH_2 _(2-fold difference). The Δ*vraSR *mutation did not affect the sensitivity to ovispirin-1, nisin, daptomycin, Pep5, LL-37 or hBD3. The same mutations in another *S. aureus *strain (RN4220) caused similar antimicrobial sensitivity differences, indicating that they are not strain specific (additional file 3).

In order to characterize further the phenotypes of the *vraDE *and *vraSR *null mutants, we subjected them to phenotype microarray (PM) analysis (Biolog). The analysis was performed with the PM11-20 sensitivity plates, which allow the testing of 960 phenotypes and the identification of increased or decreased sensitivities of the mutants as compared to the wild-type reference strain to a wide variety of non-peptide antimicrobial agents. Phenotype microarray analysis measures the reduction of tetrazolium dye (see experimental section).

**Figure 2:**
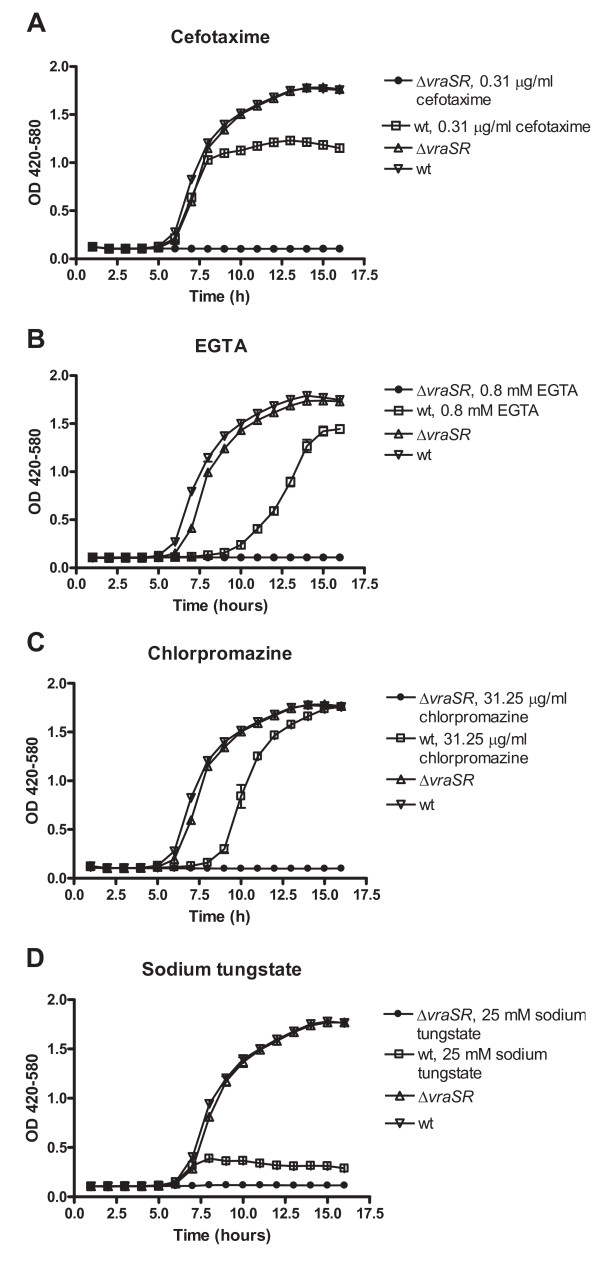
**Increased sensitivity of the Δ*vraRS *mutant to cefotaxime, EGTA, chlorpromazine and sodium tungstate**. The Δ*vraRS *mutant and the wild-type *S. aureus *Newman strain were grown either in the presence or absence of the antimicrobial agents as indicated in 150 μl of BHI medium in wells of microtiter plates in a Bioscreen for 16 hours. The optical densities of the cultures were measured every 15 minutes. The data points show the densities measured at one-hour intervals.

The Δ*vraSR *mutant was more sensitive than the wild-type strain to a number of cell wall-active antibiotics including several cephalosporins (additional files 4 and 5). This result is consistent with a similar result of a previous study on a *vraSR *null mutant of *S. aureus *N315 [33]. The PM assay also suggested enhanced sensitivity to some other antimicrobial compounds than antibiotics. We verified the growth inhibitory effects of four of these antimicrobials, cefotaxime, chlorpromazine, EGTA and sodium tungstate, by cultivating the Δ*vraSR *mutant and the wild-type *S. aureus *Newman strain in BHI medium on microtiter plates containing 0.31 μg/ml, 0.8 mM, 31.25 μg/ml and 25 mM concentrations of these antimicrobials, respectively, and measuring the optical densities of the cultures. The Δ*vraSR *mutant was unable to grow in the presence of these antimicrobials, while the wild-type strain grew significantly (Figure 2). In addition to these major differences of sensitivity, several minor differences were also observed, but we did not verify them in microtiter plate cultures by optical density measurements. The *vraSR *null mutant was resistant to macrolide antibiotics due to the *ery *gene used in the mutant construction. The PM analysis of the Δ*vraDE *mutant did not reveal any significant sensitivity differences (not shown), suggesting again a dedicated role for VraDE in bacitracin resistance.

## Discussion

A prominent feature of the CAMP transcriptomes of *S. aureus *Newman was the induction of the VraSR regulon. The regulon consists of several genes involved in cell wall-associated functions such as protein quality control, protein folding and modulation of cell wall biosynthesis [33]. The induction pattern of the VraSR-regulated genes was very similar to that observed with vancomycin-treated cells in another transcription profiling study with another *S. aureus *strain, N315 [33]. In addition to the effects on membranes, several antimicrobial peptides such as nisin, mersacidin and bacitracin inhibit cell wall biosynthesis [41,43-45]. Whether the VraSR-inducing linear CAMPs used in this study inhibit cell wall biosynthesis and cause a cell wall defect cannot be judged from our data, but this is the most likely explanation for the similar induction patterns of the VraSR-regulated genes. A transcription profiling study with a lipopeptide antibiotic daptomycin also showed up-regulation of the the VraSR cell wall regulon [46], which is probably caused by an inhibitory effect on the cell wall biosynthesis [47], although the results furthermore suggested that some of the up-regulated genes in the daptomycin transcriptome were induced by its membrane depolarization effect [46]. A similar dual effect has also been shown with human β-defensin 3 [48].

In *B. subtilis*, the *yxdLM *operon, which encodes an ABC transporter, is strongly induced by human cathelicidin LL-37 in a manner dependent on the YxdJK two-component system, but not at all by porcine protegrin PG-1 and a model antimicrobial peptide poly-L-lysine [25]. This indicates that antimicrobial peptides can be very specific in activating stress sensors and the activation mechanism may even be dependent on the mode of action, charge or structural properties of the peptides. In this study we found that the *vraDE *ones were the most strongly induced genes in cells treated with ovispirin-1-NH_2 _or dermaseptin K4-S4(1-16)-NH_2_. We could demonstrate that the high level of induction of *vraDE *with ovispirin-1-NH_2 _was dependent on the amide group at the C-terminus. The antimicrobial effects of these two variants of ovispirin-1 on wild-type *S. aureus *Newman strain were not different. The expression regulation of *vraDE *is under the control of the Aps (GraRS) sensor system, which also controls the expression of *vraFG*, encoding a putative peptide efflux pump, *dlt *operon, encoding components responsible for the D-alanylation of teichoic acids and modulation of the net negative charge of the cell wall, and *mprF*, encoding the MprF enzyme which catalyzes the lysinylation of phosphatidylglycerol and modulates the charge of the outer surface of the cell membrane [37]. It has been shown that a nine-aminoacid loop of the ApsS sensor exposed on the outer surface of the membrane interacts with CAMPs when activating the sensor [37]. Our results suggest that in linear peptides the C-terminal amide group is an important element for the activation of the ApsS sensor. In the study by Li and collaborators [37] the peptides which naturally contain an amide group at the C-terminus, indolicidin and melittin, were significantly better inducers of the *dltB *gene than those that do not have the amide such as magainin II and nisin. The *vraDE *genes were also the most strongly induced genes in the stress response to C-terminally amide-modified human cathelicidin LL-37 (our unpublished results). However, the low level of induction of *vraDE *with temporin L-NH_2 _in this study suggests that the C-terminal amide is not the only element that is recognized by the ApsS sensor and needed for the induction. Alternatively, the level of the induction of the Aps regulon might rather depend on the absence of the carboxyl group than on the presence of the amide. Mersacidin, which is a lantibiotic, but unlike nisin, does not have a free C-terminal carboxyl group due to an intramolecular thioether-ethyleneamide bridge, is a very strong inducer of *vraDE *[49] and might fit this model.

VraDE is a transporter system that is dedicated to resist bacitracin, as evidenced in this study by the testing the sensitivity of the *vraDE *null mutant(s) to 11 antimicrobial peptides or antibiotics and numerous other antimicrobial agents (PM analysis). The Δ*vraDE *mutant(s) was more susceptible to bacitracin but not to any other antimicrobial tested. Consistently, VraDE is similar to the BceAB bacitracin transport system of *B. subtilis *[34,35] and probably comprise together with VraFG an efflux pump system for combating bacitracin [37]. A recent study suggested that in *B. subtilis *bacitracin sensing and the expression of the *bceAB *genes are dependent on active bacitracin transport via BceAB and that the large periplasmic loop of the BceA permease component of the transporter may be involved in mediating the signal to the BceRS two-component system [50]. We found that bacitracin is a strong inducer of *vraDE *expression. Whether the sensing mechanism of Aps is dependent on the transport function of VraDE or VraFG, whether Aps senses linear CAMPs and bacitracin in a similar manner and whether the amide group of the side chain of glutamine in the cyclic bacitracin peptide [51] have a similar essential role in the activation, should be addressed in future studies. The C-terminal amide being in a sequence contex that is appropriate for the interaction with the sensory loop of ApsS most probably explains the differences in the induction patterns of the *vraDE*, *vraF *and *dltA *genes observed in this study. However, we can not rule out the possibility that the carpet-mode of action mechanism influences the induction, since the amide-modified carpet peptides ovispirin-1-NH_2 _and dermaseptin K4-S4(1-16)-NH_2 _were clearly stronger inducers of these genes than the amide-modified toroidal pore-forming peptide temporin L-NH_2_.

In addition to *vraDE*, SA0205 was strongly induced by CAMPs, but in this case the strongest inducer was temporin L-NH_2_. Our results indicated that SA0205, which encodes a putative cell membrane-associated peptidase, belongs to the VraSR regulon. The over 100-fold induced (qRT-PCR) SAS016 encodes a small protein with unknown function and mechanism of regulation. It was also strongly induced by vancomycin [52], suggesting that it responds to a cell wall defect. Furthermore, it was detected at both transcript and protein levels in GISA strains [53]. *S. aureus *cells try to adapt to the stress and harmful effects of CAMPs by inducing or repressing several gene systems. These responses include the induction of the *ctsR*, *dnaJ*, *dnaK*, *hrcA*, *groEL *and *groES *general stress genes and genes resisting oxidative stress (*katA*, *trxB *and SA2324). Some of these targets showed similar inductions in *S. aureus *during phagocytosis by neutrophils or when surviving within epithelial and phagocytic cells [54,55]. Notably, several amino acid biosynthesis operons and genes encoding enzymes of the citric acid cycle were also induced and genes involved in anaerobic metabolism were repressed, indicating that the cells were metabolically active and respiring aerobically. The down-regulation of virulence gene expression is partly caused by the repression of *saeRS*, but also the decreased expression of the *agr *operon affects their low expression.

*S. aureus *is a human pathogen and it can be argued that the CAMPs of animal origin may cause a different kind of transcriptional response than CAMPs of the natural host. A transcriptional analysis of responses of *S. aureus *(SG511) to human β-defensin 3 (hBD3) showed that *vraDE*, *vraSR *and SA0205 were also induced by this human CAMP [48], as was the case with the animal CAMPs in this study. The most notable difference in the transcriptomes is the very strong induction of the SA0192 gene, which encodes a protein similar to ABC transporter ATP-binding proteins, in SG511 cells exposed to hBD3. Since this gene is absent in the genome of the *S. aureus *Newman strain, it is not among the induced genes of this study. Sass and collaborators recently showed that a knockout mutation of *vraE *increases the susceptibility of *S. aureus *SG511 also to other antibiotics than bacitracin, including antimicrobial peptides hBD3, LL-37, Pep5 and nisin [48]. The defective GraRS/Aps sensory system of the strain SG511 [56] may explain the inconsistency with the result of this study.

The Δ*vraSR *mutant exhibited enhanced sensitivity to several antimicrobial agents as compared to the wild-type strains. They were more sensitive to the amide-modified ovispirin-1 but not to the non-modified one. Ovispirin-1-NH_2 _was a better activator of Aps and VraSR than ovispirin-1 and this may explain the higher resistance of the wild-type strain to the amide-modified peptide. The Δ*vraSR *mutation also decreased the MIC values of bacitracin and teicoplanin. This effect is similar to what has been observed with a Δ*vraSR *mutant of *S. aureus *N315 [33]. Our phenotype microarray analysis revealed increased sensitivity of the *S. aureus *Newman Δ*vraSR *mutant to a wide range of β-lactam antibiotics. Furthermore, we showed that Δ*vraSR *increased susceptibility to other types of antimicrobial agents such as EGTA and phenothiazines. Since EGTA is a metal-chelator, it probably harmfully affects bacterial cells and particularly severely Δ*vraSR *mutant cell by binding to divalent metal cations in the cell wall and consequently causing metal sequestration. The phenothiazines chlorpromazine and thioridazine, which are or have been used in psychiatric therapy, are potential lead compounds for the development of new cell wall-active antibiotics. It has been shown that the dosage of chlorpromazine used in psychiatry results in intracellular concentrations of this drug that are antimicrobial [57].

## Conclusion

The current rapid increase in antibiotic resistance among human pathogens coincides dangerously with a lack of novel antibiotic discovery. A promising alternative is a class of short peptides known as antimicrobial peptides, or host defense peptides, which are found among all classes of life but have yet to be widely exploited for pharmaceutical purposes. In order to identify the inducible resistance mechanism of *Staphylococcus aureus *against antimicrobial peptides and other antimicrobial agents, the bacterium was exposed to antimicrobial peptides and gene expression changes were analyzed. The analysis revealed similar gene induction patterns with cell wall-active antibiotics but also some distinctly different ones. The induction of the VraSR regulon suggests that antimicrobial peptides have an inhibitory effect on the cell wall biosynthesis. This must be verified biochemically in future studies. Parts of the signalling pathways are not specifically dedicated to CAMP defense but are regulated following the lifestyle of pathogenic bacteria which are potentially exposed to a wide diversity of environmental changes.

This study does not provide a definitive answer as to whether the mode of action of CAMPs on lipid bylayers is responsible for certain induction patterns, but this is a potential alternative. For instance in the case of the induction of the SA1217-SA1221 operon, the carpet-forming peptides ovispirin-1-NH_2 _or dermaseptin K4-S4(1-16)-NH_2 _were strong inducers whereas the pore-forming peptide temporin L-NH_2 _was unable to induce this operon. Most importantly, it was found that modifying the carboxy-terminus of a linear peptide with an amide group can modulate drastically its properties as an inducer of resistance mechanisms. This result may allow the rational design of antimicrobial agents that lack the properties to induce resistance mechanisms. Inactivating the VraSR cell wall stress management system increased susceptibility of the bacterium to a wide range of cell wall-active antibiotics but also susceptibility to several non-antibiotic antimicrobials was increased. Particularly interesting in the latter group are the established antipsychotic drugs, chlorpromazine and thioridazine, which are potential lead molecules for developing novel antibiotics.

## Methods

### Bacterial strains, plasmids and growth conditions

The bacterial strains and plasmids used are listed in Table 5. *E. coli *cells were grown in Luria-Bertani (LB) broth supplemented with ampicillin (100 μg ml^-1^) and *S. aureus *cells in BHI (Brain Heart Infusion) and TSB (Tryptone Soya Broth) media supplemented with chloramphenicol (10 μg ml^-1^) and/or erythromycin (2.5 μg ml^-1^) when needed. To determine the subinhibitory concentrations of temporin L-NH_2 _(FVQWFSKFLGRIL-NH_2_) ovispirin-1-NH_2_(KNLRRIIRKIIHIIKKYG-NH_2_) and dermaseptin K4-S4(1-16)-NH_2 _(ALWKTLLKKVLKAAAK-NH_2_), 150 μl of BHI medium was inoculated with approximately 10^5 ^*S. aureus *Newman cells in honeycomb 2 plate wells, peptides were added in two-fold dilution series, and the cultures were incubated with continuous and moderate shaking at 37°C and culture densities were measured in a Bioscreen C Microbiology reader (Growth Curves, Helsinki, Finland). The peptide treatments for the gene expression analyses were carried out in the exponential growth phase (~OD_560 _= 0.6); CAMPs were added at the final (sublethal) concentrations of 3 μM (temporin L-NH_2_), 4 μM (ovispirin-1-NH_2_) and 3 μM (dermaseptin K4-S4(1-16)-NH_2_) if not otherwise indicated. The addition of the peptides at these sublethal concentrations slowed down the growth slightly. The peptides were purchased from EZbiolab (Carmel, IN, USA) and their purity was 95%. The Minimal Inhibitory Concentration (MIC) determinations were performed by cultivating *S. aureus *Newman or RN4220 cells and their *vraSR *and/or *vraDE *null mutation derivatives in 100% (temporin L-NH_2_, ovispirin-1-NH_2_, ovispirin-1, nisin, vancomycin, bacitracin, teicoplanin, daptomycin and pentaglysine), 50% (LL-37, nisin and Pep5) or 25% (hBD3) Mueller-Hinton broth for 16-24 hours. Typically, an overnight grown culture was diluted 1/10000 and 1 ml of broth in a 15 ml sterile Falcon tube was inoculated with 10 μl of the diluted culture and cultivated at 37°C with shaking (220 rpm min^-1^). Each MIC determination was performed twice with a two-fold dilution series of the antimicrobial agents.

**Table 5:** Bacterial strains and plasmids.

**Strain/Plasmid**	**Description**	**Reference/Origin**
***E. coli***		
5-alpha	cloning strain	New England Biolabs
***S. aureus***		
Newman	ATCC25904, methicillin susceptible	[72]
RN4220	NCTC 8325-4-r, restriction negative strain	[73]
COL	methicillin resistant (MRSA)	[60]
RH7657	Newman Δ*vraSR*::ery	this study
RH7661	Newman Δ*vraDE*::ery	this study
RH7788	RN4220 Δ*vraDE*::ery	this study
RH7790	RN4220 Δ*vraSR*::ery	this study
**Plasmids**		
pGEM-3zf(+)	cloning vector, amp^r ^(*E. coli*)	Promega
pKOR1	gene replacement vector, amp^r ^(*E. coli*), chl^r ^(*S. aureus*)	[69]
pKTH3762	pGEM-3zf(+) containing the *vraSR *deletion cassette, amp^r ^(*E. coli*)	this study
pKTH3763	pKOR1 containing the *vraSR *deletion cassette, amp^r ^(*E. coli*), chl^r ^ery^r ^(*S. aureus*)	this study
pKTH3764	pGEM-3zf(+) containing the *vraDE *deletion cassette, amp^r ^(*E. coli*)	this study
pKTH3765	pKOR1 containing the *vraDE *deletion cassette, amp^r ^(*E. coli*), chl^r ^ery^r ^(*S. aureus*)	this study

### Isolation of total cellular RNA

The samples for RNA isolations were taken 10 minutes after the addition of the antimicrobial peptides or other antimicrobial agents at the cell density of OD_600 _= 0.6 or Klett 50. The cells from 1.8-2 ml of the cultures (BHI medium) were harvested by centrifugation. The supernatants were discarded and the bacterial pellets were frozen in liquid nitrogen if the RNA isolation was not conducted immediately. The control samples without peptides were treated in a similar manner. The cell pellets were resuspended in 200 μl TE-buffer (10 mM Tris-HCl, 1 mM EDTA pH 7.0) containing lysostaphin (125 μg ml^-1^) and incubated at 37°C for 10 minutes. RNA was extracted by using Roche's High Pure RNA Isolation Kit according to the manufacturer's instructions.

### Transcriptional analysis by oligo DNA microarray

The effects of the three different antimicrobial peptides on gene expression of *S. aureus *Newman were studied by using whole genome oligo-DNA microarrays. Bacteria were grown in BHI medium to the early exponential phase (OD_600 _= 0.6) and antimicrobial peptides were added at the sublethal concentrations described above. Samples were taken for RNA isolations after treating the cultures with the peptides for 10 minutes. Control cultures without peptide additions were treated similarly and in parallel. Genes with at least 2-fold induction/reduction of expression in the peptide-treated cells as compared to the control cells (non-treated) were accepted as differentially expressed genes.

The microarray was manufactured by *in situ *synthesis of 10'807, 60-mer oligonucleotide probes (Agilent, Palo Alto, CA, USA), selected as previously described [38]. It covers >98% of all ORFs annotated in strains N315 and Mu50 [58], MW2 [59] COL [60], NCTC8325 (the sequence, locus NC_007795, can be found at the National Center for Biotechnology Information [61]), USA300 [62], MRSA252 and MSSA476 [63] including their respective plasmids. Extensive experimental validation of this array has been described previously, using CGH (comparative genomic hybridization), mapping of deletion, specific PCR and quantitative RT-PCR [38,64].

Total RNA was further purified with Qiagen RNeasy mini kit and treated with DNAse following the manufacturer's recommendations. The absence of remaining DNA traces was evaluated by quantitative PCR (SDS 7700; Applied Biosystems, Framing-ham, MA) with assays specific for 16s rRNA [53,65]. Samples of 8 μg total *S. aureus *RNA were labelled by Cy-3 or Cy-5 dCTP using the SuperScript II (Invitrogen, Basel, Switzerland) following the manufacturer's instructions. Labelled products were then purified with QiaQuick columns (Qiagen). A mixture of control (without peptide) and test conditions (with peptide) was then diluted in 250 μl Agilent hybridization buffer, and hybridized at a temperature of 60°C for 17 hours in a dedicated hybridization oven (Robbins Scientific, Sunnyvale, CA, USA). The slides were washed with Agilent proprietary buffers, dried under a nitrogen flow, and scanned (Agilent, Palo Alto, CA, USA) using 100% PMT (photomultiplier tube) power for both wavelengths.

Fluorescence intensities were extracted using the Feature extraction™ software (Agilent, version 8). Local background-subtracted signals were corrected for unequal dye incorporation or unequal load of labelled product. The algorithm consisted of a rank consistency filter and a curve fit using the default LOWESS (locally weighted linear regression) method. Data consisting of three independent biological experiments were analyzed using GeneSpring 7.3 (Agilent) after per gene and per chip normalization. The statistical significance of differentially expressed genes was identified by variance analysis (ANOVA) [53,66], performed using GeneSpring, including the Benjamini and Hochberg false discovery rate correction (5%). Genes showing significant changes (P < 0.05) and at least a 2-fold induction/reduction of expression, in the peptide-treated cells as compared to the control cells (non-treated) were accepted as differentially expressed.

### Quantitative real-time RT-PCR

cDNA was synthesized with 1 μg of total cellular RNA by using a High Capacity cDNA Archive Kit (Applied Biosystems) with an additional step of DNase I (Roche) treatment [67] The quantitative real-time PCR was performed as previously described [25] by using specific primer pairs and a SYBR Green PCR system kit (Applied Biosystems). The primers (additional file 6) were purchased from TAG Copenhagen. The amplification reactions and detection of PCR products were performed with a 7500 real time PCR system (Applied Biosystems). The cDNA values were normalized with the value of *gyrA*.

### Construction of the vraDE and vraSR null mutants

In order to construct null mutants of the *vraDE *and *vraSR *operons, about 1 kb DNA fragments of both the upstream and downstream regions of the target operons were PCR amplified. The template DNA was *S. aureus *chromosome either from the strain Newman (*vraSR*) or COL (*vraDE*). The primers used in the PCR reactions are shown in additional file 6. The upstream fragments digested with *Pst*I and *Sal*I and the downstream fragments digested with *Bam*HI and *Eco*RI/*Sac*I were ligated with a PCR-amplified DNA fragment containing the erythromycin resistance gene of pMUTIN4 [68]. The resulting *vraDE *and *vraSR *inactivation cassettes were inserted into the pGEM3Zf(+) plasmid vector between the *Pst*I and *Eco*RI/*Sac*I sites, followed by transformation of competent *E. coli *5-alpha (NEB) cells with the ligated DNA. The inactivation cassettes were then PCR amplified by using the pGEM3Zf(+) constructs as PCR templates and specific oligonucleotides, containing the sequences of attB1 and attB2 sites, as PCR primers. The amplified fragments were inserted into the pKOR1 gene replacement plasmid in a recombination reaction with Clonase (Invitrogen) as previously described [69]. The resulting pKOR1-inactivation cassette constructs were used to transform electrocompetent *S. aureus *RN4220 cells by electroporation [70] and transformants were selected by cultivating them on BHI agar plates supplemented with chloramphenicol (10 μg ml^-1^) and erythromycin (2.5 μg ml^-1^) at 30°C. The gene replacement plasmids were isolated from the RN4220 transformants and subsequently electroporated into *S. aureus *Newman cells. The mutant construction was performed as described [69]. Briefly, *S. aureus *Newman or RN4220 cells harbouring the gene replacement plasmids were cultivated in TSB medium overnight at 43°C, a non-permissive temperature for the replication of pKOR1 and its derivative plasmids, to force the plasmids to integrate into the chromosome by a Campbell-type recombination at a flanking region of the target gene. The resulting merodiploid cells were cultivated on TSA plates containing 100 ng ml^-1 ^anhydrotetracyclin but not chloramphenicol to induce pKOR1-encoded *secY *antisense transcripts and counter-select cells which via homologous recombination between the gene duplicates had lost the pKOR1. The presence of erythromycin (2.5 μg ml^-1^) on the plates allowed the selection of the mutants. The mutants were verified by PCR with appropriate primers (additional file 6).

### Phenotype microarray (PM) analysis of the vraDE and vraSR null mutants

The PM analysis was carried out by using the service of Biolog (Hayward, CA, USA). We wanted to identify antimicrobial agents and stress conditions which are more harmful to the mutant cells than the wild-type *S. aureus *Newman cells. Therefore, the analysis was performed only with sensitivity panels 11-20, which allowed the testing of 960 stress conditions and 240 different antimicrobial agents on ten 96-well plates. The method has been described more thoroughly in a previous study [71]. Briefly, the growth of bacteria in a well of the PM panels can be quantitated since growing, metabolically-active bacteria reduce the tetrazolium indicator dye and blue color is formed. Bacteria were grown in BHI medium for 24 hours in an OmniLog instrument. The color changes of the redox dye in the cultures were compared (mutant *versus *wild type). The formation of the reduced tetrazolium was recorded either as a green tracing (mutant) or a red tracing (wild-type strain). The OmniLog PM bioinformatics software was used to overlay the color-coded images of the kinetics of the tetrazolium reduction and compare and quantify them. Each strain was analyzed twice and consensus images were obtained showing the differences in antimicrobial sensitivity that were detected in both PM runs.

## Authors' contributions

Experimental planning: VPK. Performed DNA microarrays and data analysis: PF JS MP VPK. Performed qRT-PCR measurements: MP HLH. Constructed mutants: MP VPK. Performed antimicrobial sensitivity measurements: MP VPK VS HGS. Wrote the paper: MP PF VPK.

## Supplementary Material

Additional file 1 **Genes and operons induced by several cationic antimicrobial peptides**. The table shows the genes induced at least 2-fold by several cationic antimicrobial peptides. Click here for file

Additional file 2 **Genes and operons down-regulated by several cationic antimicrobial peptides**. The table shows the genes down-regulated by several cationic antimicrobial peptides. Click here for file

Additional file 3 **Antimicrobial sensitivity of *S. aureus *RN4220 and its Δ*vraDE *and Δ*vraSR *mutant derivatives**. The table shows MIC values of antimicrobial peptides and other antimicrobial agents against *S. aureus *RN4220 and its Δ*vraDE *and Δ*vraSR *mutant derivatives. Click here for file

Additional file 4 **Antimicrobial sensitivities of RH7657 (vraSR::ery) and RH7603 (S. aureus Newman) were compared by using phenotype microarrays**. Scatter plots of parameter values from two replicates of the PM analysis are shown in the two uppermost panels. The three other panels show overlaid color-coded images of tetrazolium reduction kinetics (mutant versus wild type) over all wells in the two runs of the analysis and their consensus. Click here for file

Additional file 5 **Decreased reduction of tetrazolium in *vraSR *null mutant treated with antimicrobial agents as compared to the wild-type *S. aureus *Newman strain**. The gene list was obtained from the consensus of the two independent phenotype microarray analyses shown in additional file 4. Click here for file

Additional file 6 **Specific primer pairs for qRT-PCR and PCR**. The table shows the primers used in this study. Click here for file
