# Supplementary material for: Transcriptome analysis of the responses of Staphylococcus aureus to antimicrobial peptides and characterization of the roles of vraDE and vraSR in antimicrobial resistance
Source: BMC Genomics. 2009 Sep 14;10:429. doi: 10.1186/1471-2164-10-429 (PMC2748101; doi:10.1186/1471-2164-10-429)
Supplement: Additional file 1 — Genes and operons induced by several cationic antimicrobial peptides. The table shows the genes induced at least 2-fold by several cationic antimicrobial peptides. [file 1471-2164-10-429-S1.doc]

| Table S1: Genes and operons induced by several cationic antimicrobial peptides. | | | | | | | |
| --- | --- | --- | --- | --- | --- | --- | --- |
| Gene ID | Gene name | Induced with* | Fold induction | Known regulation† | Predicted localization of protein§ | Protein/Similarity | FC¶ |
| **SA0011** |  | **T,O** | **3.5, 2.1** |  | **c** | **Similar to homoserine-o-acethyltransferase** | **E** |
| **SA0122** | ***butA*** | **T,O** | **3.2, 2.1** | **VCM** | **c** | **Acetoin reductase** | **F** |
| **SA0162** | ***aldA*** | **T,O** | **2.8, 2.5** | **VCM** | **c** | **Aldehyde dehydrogenase homologue** | **F** |
| **SA0172** |  | **T,O** | **2.7, 2.3** | **VCM** | **m (12)** | **Similar to integral membrane protein LmrP** | **A,G** |
| **SA0205** |  | **T,O,D** | **17.2, 11.2, 6.2** | **VCM** | **m (1)** | **Similar to lysostaphin precursor** | **K** |
| **SA0241** | ***ispD*** | **T,O** | **2.1, 2.0** |  | **c** | **Similar to 4-diphosphocytidyl-2C-methyl-D-erythritol synthase** | **F** |
| **SA0242** |  | **T,O** | **2.3, 2.1** |  | **c** | **Similar to xylitol dehydrogenase** | **F** |
| **SA0294** |  | **T,O** | **2.0, 2.0** |  | **m (12)** | **Similar to branched-chain amino acid uptake carrier** | **G** |
| **SA0298** |  | **T,O** | **2.0, 2.2** |  | **m (9)** | **Similar to regulatory protein PfoR** | **K** |
| **SA0335** |  | **T,O** | **2.1, 2.1** |  | **m (1)** | **Component of the twin-arginine translocation pathway** | **G** |
| **SA0336** |  | **T,O,D** | **2.2, 2.1, 2.1** |  | **c** | **Hypothetical protein** | **K** |
| **SA0342** |  | **O,D** | **2.2, 2.2** |  | **c** | **Acetyl-CoA C-acetyltransferase homologue** | **F** |
| **SA0358** |  | **O,D** | **2.4, 2.4** |  | **m (1)** | **Hypothetical protein** | **K** |
| **SA0343** |  | **T** | **3.0** | **VCM** | **c** | **Hypothetical protein** | **K** |
| **SA0344** | ***metE*** | **T,O** | **4.5, 2.3** | **VCM** | **c** | **5-methyltetrahydropteroyltriglutamate-homocysteine methyltransferase** | **E** |
| **SA0345** |  | **T** | **3.3** |  | **c** | **Bifunctional homocysteine S-methyltransferase/5,10-methylenetetrahydrofolate reductase protein** | **E** |
| **SA0346** |  | **T,O** | **2.3, 2.9** | **VCM** | **c** | **Similar to cystathione beta-lyase** | **E,F** |
| **SA0428** |  | **T,O,D** | **3.6, 2.4, 2.3** |  | **m (8)** | **Hypothetical protein** | **K** |
| **SA0430** | ***gltB*** | **T,O** | **4.4, 2.5** |  | **c** | **Glutamate synthase large subunit** | **E** |
| **SA0431** | ***gltD*** | **T** | **3.6** | **VCM** | **c** | **NADH-glutamate synthase small subunit** | **E** |
| **SA0480** | ***ctsR*** | **T,O** | **4.2, 4.2** |  | **c** | **Transcription repressor of class III stress genes homologue** | **C** |
| **SA0481** |  | **T,O** | **4.1, 4.0** | **VCM** | **c** | **Conserved hypothetical protein** | **K** |
| **SA0482** |  | **T,O** | **4.2, 4.3** | **VCM** | **c** | **Putative ATP:guanido phosphotransferase** | **F** |
| **SA0483** | ***clpC*** | **T,O** | **3.1, 3.0** | **VCM** | **c** | **Endopeptidase** | **D** |
| **SA0512** |  | **T,O** | **2.8, 2.1** | **VCM** | **c** | **Branched-chain amino acid aminotransferase** | **E** |
| **SA0513** |  | **T,O** | **3.3, 2.2** |  | **c** | **Conserved hypothetical protein** | **K** |
| **SA0526** |  | **T,D** | **2.2, 2.0** |  | **c** | **Hypothetical protein** | **K** |
| **SA0591** |  | **T,O,D** | **3.0, 3.3, 2.1** | **VCM** | **m (7)** | **Hypothetical protein** | **K** |
| **SA0621** |  | **T,O** | **2.7, 2.2** |  | **m (6)** | **Conserved hypothetical protein (interacts with FtsH?)** | **K** |
| **SA0639** |  | **T,O** | **2.0, 2.0** |  | **m (5)** | **Similar to ABC transporter required for expression of cytochrome bd** | **G** |
| **SA0640** |  | **T** | **2.1** |  | **m (5)** | **Similar to ABC transporter required for expression of cytochrome bd** | **G** |
| **SA0677** |  | **T,O** | **3.6, 2.1** | **VCM** | **c** | **Similar to choline transport ATP-binding protein** | **G** |
| **SA0678** |  | **T** | **2.2** | **VCM** | **m (6)** | **Similar to choline transporter** | **G** |
| **SA0716** | ***lgt*** | **T,O** | **2.0, 2.0** |  | **m (5)** | **Prolipoprotein diacylglyceryl transferase** | **I** |
| **SA0719** | ***trxB*** | **T,O** | **2.1, 2.0** |  | **c** | **Thioredoxine reductase** | **F** |
| **SA0780** |  | **T,O** | **2.9, 2.2** |  | **m (4)** | **Similar to hemolysin** | **K** |
| **SA0781** |  | **T,O** | **3.1, 2.1** |  | **c** | **Similar to 2-nitropropane dioxygenase** | **F** |
| **SA0817** |  | **T,O** | **5.0, 2.7** |  | **c** | **Similar to NADH-dependent flavin oxidoreductase** | **F** |
| **SA0824** |  | **T,O** | **2.3, 2.7** | **VCM** | **m (5)** | **Uncharacterized conserved protein** | **K** |
| **SA0825** | ***spsA*** | **T,O,D** | **2.6, 3.5, 2.0** | **VraSR,VCM** | **m (1)** | **Type-1 signal peptidase** | **D,G** |
| **SA0826** | ***spsB*** | **T,O,D** | **2.1, 2.7, 2.0** | **VCM** | **m (1)** | **Type-1 signal peptidase 1B** | **D,G** |
| **SA0833** |  | **T,O** | **2.0, 2.1** |  | **c** | **Hypothetical protein** | **K** |
| **SA0835** | ***clpB*** | **T,O** | **3.9, 4.1** | **VCM** | **c** | **ClpB chaperone homologue** | **A** |
| **SA0845** | ***oppB*** | **T,O** | **4.3, 2.0** | **VCM** | **m (6)** | **Oligopeptide transport system permease**  **protein** | **G** |
| **SA0846** |  | **T,O** | **4.9, 2.0** | **VCM** | **m (6)** | **Similar to oligopeptide transport system permease protein OppC** | **G** |
| **SA0847** | ***oppD*** | **T,O** | **4.5, 2.0** | **VCM** | **c** | **Oligopeptide transport system ATP-binding protein OppD homologue** | **G** |
| **SA0848** | ***oppF*** | **T** | **4.8** | **VCM** | **c** | **Oligopeptide transport system ATP-binding protein OppF homologue** | **G** |
| **SA0849** |  | **T** | **3.9** | **VCM** | **m (lp)** | **Similar to peptide binding protein OppA** | **G** |
| **SA0884** |  | **T,O** | **2.4, 2.6** |  | **c** | **Lipoate-protein ligase homologue** | **I** |
| **SA0902** |  | **T,O** | **2.2, 2.2** |  | **c** | **Hypothetical protein/HisC homologue** | **K** |
| **SA0903** |  | **T,O,D** | **2.1, 4.1, 2.6** |  | **m (10)** | **Conserved hypothetical protein** | **K** |
| **SA0914** |  | **T,O,D** | **2.3, 2.5, 2.0** | **VraSR,VCM** | **c** | **Similar to chitinase B** | **K** |
| **SA0962** |  | **T,O,D** | **2.4, 2.8, 2.4** |  | **m (9)** | **Conserved hypothetical protein** | **K** |
| **SA0997** | ***murI*** | **T** | **2.2** |  | **c** | **Glutamate racemase** | **E** |
| **SA0998** |  | **T,O** | **2.4, 2.0** |  | **c** | **Conserved hypothetical protein** | **K** |
| **SA0999** |  | **T,O** | **2.3, 2.1** |  | **c** | **Conserved hypothetical protein** | **K** |
| **SA1164** | ***dhoM*** | **T,O** | **5.2, 2.8** | **VCM** | **c** | **Homoserine dehydrogenase** | **E** |
| **SA1165** | ***thrC*** | **T,O** | **5.5, 3.2** | **VCM** | **c** | **Threonine synthase** | **E** |
| **SA1166** | ***thrB*** | **T,O** | **3.6, 2.2** |  | **c** | **Homoserine kinase homologue** | **E** |
| **SA1170** | ***katA*** | **T,O** | **4.3, 2.6** |  | **c** | **Catalase** | **F** |
| **SA1184** | ***citB*** | **T,O,D** | **2.5, 2.4, 2.0** | **VCM** | **c** | **Aconitate hydratase** | **F** |
| **SAS043** |  | **T,O** | **2.3, 2.0** |  | **c** | **Hypothetical protein** | **K** |
| **SA1192** |  | **T,O** | **2.1, 2.1** |  | **m (8)** | **Hypothetical protein** | **K** |
| **SA1216** |  | **T,O** | **3.8, 2.4** |  | **c** | **Similar to oligoendopeptidase** | **D** |
| **SA1217** |  | **O,D** | **3.0, 2.4** |  | **c** | **Hypothetical protein, similar to negative regulator PhoU** | **C** |
| **SA1218** | ***pstB*** | **D** | **2.9** |  | **c** | **Phosphate ABC transporter, ATP-binding protein** | **G** |
| **SA1219** |  | **O,D** | **5.4, 4.2** |  | **m (6)** | **Similar to phosphate ABC transporter** | **G** |
| **SA1220** |  | **O,D** | **2.3, 2.1** |  | **m (6)** | **Similar to phosphate ABC transporter** | **G** |
| **SA1221** |  | **O,D** | **3.1, 2.8** |  | **m (lp)?** | **Phosphate-binding lipoprotein** | **G** |
| **SA1225** | ***lysC*** | **T,O** | **5.8, 8.3** |  | **c** | **Aspartokinase II** | **E** |
| **SA1226** | ***asd*** | **T,O** | **7.4, 9.3** | **VCM** | **c** | **Aspartate semialdehyde dehydrogenase** | **E** |
| **SA1227** | ***dapA*** | **T,O** | **8.3, 9.6** | **VCM** | **c** | **Dihydrodipicolinate synthase** | **E** |
| **SA1228** | ***dapB*** | **T,O** | **8.3, 8,4** | **VCM** | **c** | **Dihydrodipicolinate reductase** | **E** |
| **SA1229** | ***dapD*** | **T,O** | **6.6, 6.5** | **VCM** | **c** | **Tetrahydrodipicolinate acetyltransferase** | **E** |
| **SA1230** |  | **T,O** | **5.6, 4.8** | **VCM** | **c** | **Hippurate hydrolase** | **D** |
| **SA1231** |  | **T,O** | **6.2, 4.6** |  | **c** | **Similar to alanine racemase** | **E** |
| **SA1232** | ***lysA*** | **T,O** | **6.6, 5.1** |  | **c** | **Diaminopimelate decarboxylase** | **E** |
| **SA1235** |  | **T,O,D** | **2.4, 2.1, 2.6** |  | **c** | **Hypothetical protein** | **K** |
| **SA1237** |  | **T,O** | **2.0, 2.2** |  | **m (2)** | **Similar to 5-bromo-4-chloroindolyl phosphate hydrolysis protein XpaC** | **F** |
| **SA1253** | ***ctpA*** | **T** | **2.2** | **VraSR,VCM** | **m (1)** | **Probable carboxy-terminal processing proteinase ctpA** | **D** |
| **SA1254** |  | **T,O** | **3.1, 3.5** | **VraSR,VCM** | **c** | **Hypothetical protein** | **K** |
| **SA1255** |  | **T,O** | **2.6, 2.9** | **VraSR,VCM** | **c** | **PTS system, glucose-specific enzyme II, A component** | **F,G** |
| **SA1256** |  | **T,O** | **3.0, 3.2** | **VCM** | **c** | **Methionine sulfoxide reductase B** | **E** |
| **SA1257** | ***msrA1*** | **T,O** | **2.3, 2.6** | **CWA** | **c** | **Peptide methionine sulfoxide reductase A** | **E** |
| **SA1406** |  | **T,O** | **2.4, 2.9** |  | **c** | **Conserved hypothetical protein** | **K** |
| **SA1407** |  | **T,O** | **2.6, 3.3** |  | **c** | **Conserved hypothetical protein** | **K** |
| **SA1408** | ***dnaJ*** | **T,O** | **2.2, 2.6** |  | **c** | **DnaJ protein** | **A** |
| **SA1409** | ***dnaK*** | **T,O** | **2.5, 2.9** |  | **c** | **DnaK protein** | **A** |
| **SA1411** | ***hrcA*** | **T,O** | **2.0, 2.3** |  | **c** | **Heat-inducible transcriptional repressor** | **C** |
| **SA1476** |  | **T,O,D** | **4.2, 5.3, 3.3** | **VraSR,VCM** | **e** | **Hypothetical protein** | **K** |
| **SA1477** |  | **T,O,D** | **5.5, 6.8, 3.8** | **VCM** | **m (2)** | **Hypothetical protein** | **K** |
| **SA1517** | ***citC*** | **T,O,D** | **4.9, 5.8, 3.1** | **VCM** | **c** | **Isocitrate dehydrogenase** | **F** |
| **SA1518** | ***citZ*** | **T,O,D** | **6.0, 6.7, 3.6** | **VCM** | **c** | **Citrate synthase** | **F** |
| **SA1544** |  | **T,O** | **4.1, 2.5** | **VCM** | **c** | **Similar to soluble hydrogenase 42 kD subunit** | **E** |
| **SA1545** | ***serA*** | **T,O** | **4.6, 2.5** | **VCM** | **c** | **D-3-phosphoglycerate dehydrogenase** | **E** |
| **SA1546** |  | **T,O** | **2.2, 2.0** | **VCM** | **c** | **Hypothetical protein** | **K** |
| **SA1549** | ***htrA*** | **T,O,D** | **2.9, 3.0, 2.0** | **VraSR,VCM** | **m (1)** | **Similar to serine proteinase Do, heat-shock protein HtrA** | **A** |
| **SA1594** |  | **T,O** | **2.2, 2.1** |  | **c** | **Hypothetical protein** | **K** |
| **SA1599** |  | **T,O** | **3.2, 2.4** |  | **c** | **Similar to transaldolase** |  |
| **SA1654** | ***ecsB*** | **T,O** | **2.3, 2.3** |  | **m (10)** | **ABC transporter EcsB homologue** | **K** |
| **SA1655** | ***ecsA*** | **T,O** | **3.0, 3.0** |  | **c** | **ABC transporter EcsA homologue** | **K** |
| **SA1659** | ***prsA*** | **T,O,D** | **3.2, 4.5, 2.9** | **VraSR,VCM** | **m (lp)** | **Peptidyl-prolyl cis/trans isomerase homologue** | **A** |
| **SA1679** |  | **T,O** | **2.9, 2.3** |  | **c** | **Similar to D-3-phosphoglycerate dehydrogenase** | **E** |
| **SA1680** |  | **T** | **2.3** |  | **c** | **Conserved hypothetical protein** | **K** |
| **SA1691** | ***sgtB*** | **T,O** | **2.0, 2.9** | **VraSR,VCM** | **m (1)** | **Similar to penicillin-binding protein 1A/1B** | **H** |
| **SA1700** | ***vraR*** | **T,O,D** | **3.2, 5.0, 2.8** | **VraSR,VCM** | **c** | **Two-component response regulator** | **B** |
| **SA1701** | ***vraS*** | **T,O,D** | **3.9, 5.3, 2.5** | **VraSR,VCM** | **m (2)** | **Two-component sensor histidine kinase** | **B** |
| **SA1702** |  | **T,O,D** | **4.3, 5.7, 2.6** | **VraSR,VCM** | **m (4)** | **Conserved hypothetical protein** | **K** |
| **SA1703** |  | **T,O,D** | **3.7, 4.2, 2.1** | **VraSR,VCM** | **c** | **Hypothetical protein** | **K** |
| **SA1706** |  | **T,O,D** | **2.0, 2.0, 2.1** |  | **c** | **Hypothetical protein** | **K** |
| **SA1710** |  | **T,O,D** | **2.2, 2.4, 2.1** |  | **c** | **Similar to DNA polymerase III, alpha chain PolC type** | **C** |
| **SA1820** |  | **T,O,D** | **3.6, 3.0, 2.2** |  | **c** | **Similar to bacteriophage terminase small subunit** | **K** |
| **SA1821** |  | **T,O** | **3.1, 2.8** |  | **c** | **Hypothetical protein** | **K** |
| **SA1836** | ***groEL*** | **T,O,D** | **3.8, 4.3, 2.2** |  | **c** | **GroEL protein** | **A** |
| **SA1837** | ***groES*** | **T,O,D** | **3.2, 3.6, 2.4** |  | **c** | **GroES protein** | **A** |
| **SA1858** | ***ilvD*** | **T,O,D** | **4.9, 2.5, 2.1** |  | **c** | **Dihydroxy-acid dehydratase** | **E** |
| **SA1859** | ***ilvB*** | **T,O** | **9.0, 2.9** |  | **c** | **Acetolactate synthase large subunit** | **E** |
| **SA1860** |  | **T,O,D** | **15.7, 4.3, 2.1** |  | **c** | **Similar to acetolactate synthase small subunit** | **E** |
| **SA1861** | ***ilvC*** | **T,O** | **12.1, 3.2** |  | **c** | **Ketol-acid reductoisomerase** | **E** |
| **SA1862** | ***leuA*** | **T,O,D** | **13.5, 3.3, 2.1** |  | **c** | **2-isopropylmalate synthase** | **E** |
| **SA1863** | ***leuB*** | **T,O,D** | **9.7, 4.2, 2.2** |  | **c** | **3-isopropylmalate dehydrogenase** | **E** |
| **SA1864** | ***leuC*** | **T,O** | **9.9, 3.2** |  | **c** | **3-isopropylmalate isomerase large subunit** | **E** |
| **SA1865** | ***leuD*** | **T** | **3.4** |  | **c** | **3-isopropylmalate dehydratase small subunit** | **E** |
| **SA1866** | ***ilvA*** | **T** | **2.6** |  | **c** | **Threonine dehydratase** | **E** |
| **SA1934** |  | **T,O** | **2.3, 2.1** |  | **c** | **Hypothetical proteon** | **K** |
| **SA1935** | ***hmrA*** | **T,O** | **2.6, 2.2** |  | **c** | **Similar to amidase (HmrA)** | **K** |
| **SA1971** |  | **T,O,D** | **2.2, 2.5, 2.2** | **VCM** | **m (4)** | **Hypothetical protein** | **K** |
| **SA1988** |  | **T** | **2.4** |  | **c** | **Similar to alginate lyase** | **F** |
| **SA1989** |  | **T,O** | **2.4, 2.4** |  | **c** | **Similar to quinone oxidoreductase** | **F** |
| **SA1990** |  | **T,O,D** | **3.4, 2.6, 2.2** |  | **e/m (1)?** | **Conserved hypothetical protein** | **K** |
| **SA2049** |  | **T,O** | **2.0, 2.4** |  | **m (4)** | **Hypothetical protein** | **K** |
| **SA2103** |  | **T,O** | **2.5, 2.6** | **VraSR,VCM** | **m (1)/e?** | **Similar to lyt divergon expression attenuator LytR** | **C** |
| **SA2113** |  | **T,O,D** | **2.2, 3.7, 2.2** | **VraSR,VCM** | **c** | **Hypothetical protein** | **K** |
| **SA2138** |  | **T,O** | **2.0, 2.1** |  | **c** | **Hypothetical protein** | **K** |
| **SA2144** |  | **T,O** | **2.2, 2.1** |  | **c** | **Similar to transcriptional regulator (TetR/AcrR family)** | **C** |
| **SA2146** | ***tcaA*** | **T,O** | **2.5, 2.6** | **VraSR,VCM** | **m (1)** | **TcaA protein** | **A** |
| **SA2161** |  | **T,O** | **2.3, 2.9** |  | **c** | **Similar to attachment to host cells and virulence** | **K** |
| **SA2162** |  | **T,O** | **2.4, 2.7** |  | **c** | **Similar to thioredoxin reductase** | **F** |
| **SA2221** |  | **T,O** | **2.4, 3.2** | **VraSR,VCM** | **m (1)** | **Hypothetical protein** | **K** |
| **SA2248** |  | **T,O** | **2.8, 2.3** |  | **m (1)/e?** | **Similar to glutamate synthase (ferredoxin)** | **E,F** |
| **SA2297** |  | **T,O** | **2.3, 2.7** | **VraSR,VCM** | **c** | **Similar to GTP-pyrophosphokinase** | **F** |
| **SA2301** |  | **T,O,D** | **2.3, 2.2, 2.1** |  | **m (5)** | **Similar to alkaline phosphatase** | **K** |
| **SA2304** | ***fbp*** | **T,O** | **4.8, 2.4** | **VCM** | **c** | **Fructose-bisphosphatase** | **F** |
| **SA2311** |  | **T,O** | **2.2, 2.0** |  | **c** | **Similar to NAD(P)H-flavin oxidoreductase** | **F** |
| **SA2323** |  | **T,O** | **2.8, 2.1** |  | **c** | **Hypothetical protein** | **K** |
| **SA2324** |  | **T,O,D** | **4.9, 2.6, 2.0** |  | **c** | **Similar to thioredoxin** | **I** |
| **SA2325** |  | **T,O** | **3.3, 2.0** |  | **c** | **Conserved hypothetical protein** | **K** |
| **SA2342** |  | **T,O** | **2.2, 2.0** |  | **c** | **Similar to O-acetyltransferase** | **F** |
| **SA2343** |  | **T,O,D** | **4.3, 4.3, 2.7** | **VraSR,VCM** | **e** | **Hypothetical protein** | **K** |
| **SA2346** |  | **T,O** | **2.6, 3.0** | **VCM** | **c** | **Similar to D-specific D-2-hydroxyacid dehydrogenase ddh homologue** | **E** |
| **SA2347** |  | **T,O** | **2.7, 2.4** | **VCM** | **c** | **Similar to aspartate aminotransferase** | **E** |
| **SA2397** |  | **T,O** | **3.5, 2.3** | **VCM** | **c** | **Similar to pyridoxal-phosphate dependent aminotransferase** | **F** |
| **SA2464** | ***hisI*** | **T,O** | **15.6, 2.1** | **VCM** | **c** | **Histidine biosynthesis bifunctional protein HisIE** | **E** |
| **SA2465** | ***hisF*** | **T,O** | **32.6, 3.5** | **VCM** | **c** | **Cyclase-like protein HisF** | **E** |
| **SA2466** |  | **T,O** | **28.9, 3.2** | **VCM** | **c** | **Similar to phosphoribosylformimino-5-aminoimidazole carboxamide ribotide isomerase** | **E** |
| **SA2467** | ***hisH*** | **T,O** | **40.1, 4.3** | **VCM** | **c** | **Amidotransferase HisH** | **E** |
| **SA2468** | ***hisB*** | **T,O** | **33.8, 3.9** | **VCM** | **c** | **Imidazoleglycerol-phosphate dehydratase** | **E** |
| **SA2469** |  | **T,O** | **37.6, 4.2** |  | **c** | **Similar to histidinol-phosphate transaminase** | **E** |
| **SA2470** |  | **T,O** | **17.6, 4.4** | **VCM** | **c** | **Similar to histidinol dehydrogenase** | **E** |
| **SA2471** | ***hisG*** | **T,O** | **27.2, 4.9** | **VCM** | **c** | **ATP phosphoribosyltransferase** | **E** |
| **SA2472** |  | **T,O** | **12.0, 3.3** |  | **c** | **Similar to ATP phosphoribosyltransferase regulatory subunit** | **F** |
| **SA2473** |  | **T** | **3.2** |  | **c** | **Hypothetical protein** | **K** |
| **SA2474** |  | **T** | **5.2** | **VCM** | **c** | **Conserved hypothetical protein** | **K** |
| **SA2475** |  | **T** | **6.9** | **VCM** | **m (5)** | **Conserved hypothetical protein/ABC transporter** | **G** |
| **SA2476** |  | **T,O** | **9.0, 2.7** | **VCM** | **c** | **Similar to cation ABC transporter (ATP-binding protein)** | **G** |
| **SA2477** |  | **T,O** | **6.7, 3.1** |  | **m (5)** | **Conserved hypothetical protein** | **K** |
| **SA2478** |  | **T,O** | **7.4, 2.8** |  | **c** | **Conserved hypothetical protein** | **K** |
| **SA2481** |  | **T,O,D** | **2.1, 3.1, 2.7** |  | **c** | **Conserved hypothetical protein** | **K** |
| **SA2487** |  | **T,O** | **2.4, 2.0** |  | **m (10)** | **Similar to RarD protein** | **K** |
| **SA2490** |  | **T,O** | **2.5, 2.4** |  | **c** | **Similar to N-hydroxyarylamine O-acetyltransferase** | **F** |
| **SA2492** | ***vraD*** | **T,O,D** | **8.2, 32.4, 17.2** | **VCM** | **c** | **Similar to ABC transporter** | **G** |
| **SA2493** | ***vraE*** | **T,O,D** | **3.4, 11.8, 6.2** | **VCM** | **m (10)** | **Similar to ABC transporter (permease)** | **G** |
| **SAS016** |  | **T,O,D** | **5.0, 7.4, 5.4** | **VCM** | **?** | **Hypothetical protein** | **K** |
| **SAS051** |  | **T,O** | **2.0, 2.3** | **VCM** | **c** | **Hypothetical protein** | **K** |
| ***: T, temporin L-NH_2_; O, ovispirin-1-NH_2_; D, dermaseptin K4-S4(1-16)-NH_2_;**  †**: VCM, vancomycin inducible; CWA, cell wall-antibiotic inducible VraSR, belongs to the VraSR regulon;**  **§: m, predicted membrane protein, the number of putative transmembrane segments is in parenthesis; c, predicted cytoplasmic protein; e, exported protein; lp, lipoprotein;**  **¶: FC, Functional category; A, Stress combating mechanisms, protein folding, chaperon function; B, Signal transduction (two-component systems); C, Transcription, transcription regulation, replication, integration,** **nucleic acid metabolism, translation, ribosomes; D, Proteolysis, peptide cleavage; E, Amino acid metabolism and biogenesis; F, Other metabolic/biosynthetic functions (e.g. energy metabolism and carbohydrate metabolism); G, Molecule/ion uptake, export, protein secretion; H, Cell wall structure, biogenesis, hydrolysis and modification, cell division; I, Protein modification; J, Virulence and pathogenesis; K, Functionally unknown proteins, hypothetical proteins with conserved domains, hypothetical proteins.** | | | | | | | |
