# Supplementary material for: Transcriptome analysis of the responses of Staphylococcus aureus to antimicrobial peptides and characterization of the roles of vraDE and vraSR in antimicrobial resistance
Source: BMC Genomics. 2009 Sep 14;10:429. doi: 10.1186/1471-2164-10-429 (PMC2748101; doi:10.1186/1471-2164-10-429)
Supplement: Additional file 2 — Genes and operons down-regulated by several cationic antimicrobial peptides. The table shows the genes down-regulated by several cationic antimicrobial peptides. [file 1471-2164-10-429-S2.doc]

| **Table S2: Genes and operons down-regulated by several cationic antimicrobial peptides.** | | | | | | | |
| --- | --- | --- | --- | --- | --- | --- | --- |
| **Gene ID** | **Gene name** | **Down-regulated with*** | **Fold reduction** | **Known regulation†** | **Predicted localization of protein§** | **Protein/Similarity** | **FC¶** |
| SA0022 |  | T,O,D | 2.3, 2.1, 2.2 |  | e (w) | Similar to 5'-nucleotidase | C |
| SA0107 | *spa* | T,O,D | 3.0, 3.1, 3.1 | Agr-/SarA- | e (w) | Immunoglobulin G binding protein A precursor | J |
| SA0108 | *sarH1* | T,O,D | 3.6, 3.5, 3.0 |  | c? | Staphylococcal accessory regulator A homologue | A |
| SA0131 | *pnp* | T,O,D | 3.7, 3.8, 2.5 |  | c | Purine nucleoside phosphorylase | F |
| SA0132 |  | T,O | 2.2, 2.3 |  | m (14) | Similar to tetracyclin resistance protein | K |
| SA0175 |  | T,O | 2.8, 2.1 |  | m (3) | Conserved hypothetical protein | K |
| SA0183 | *glcA* | T,O,D | 2.6, 2.1, 2.1 |  | m (10) | PTS enzyme II (EC 2.7.1.69), glucose-specific, factor IIA homologue | F,G |
| SA0213 |  | T,O,D | 3.9, 5.5, 2.2 |  | m (3) | Hypothetical protein | K |
| SA0218 | *pflB* | T,O,D | 12.7, 25.5, 11.1 |  | c | Formate acetyltransferase | F |
| SA0219 | *pflA* | T,O,D | 11.9, 25.0, 11.3 |  | c | Formate acetyltransferase activating enzyme | I |
| SA0230 |  | T,O,D | 2.1, 3.6, 2.5 |  | c | Conserved hypothetical protein | K |
| SA0231 |  | T,O,D | 2.1, 3.1, 2.7 |  | c | Similar to flavohemoprotein | K |
| SA0232 | *ictE* | T,O,D | 4.8, 2.6, 3.2 |  | c | L-lactate dehydrogenase | F |
| SA0249 | *scdA* | T,O,D | 5.1, 5.4, 3.9 |  | c | Cell division and morphogenesis-related protein | H |
| SA0252 | *lrgA* | T,O,D | 4.0, 3.3, 2.0 | SaeRS+ | m (4) | Murein hydrolase regulator LrgA | H |
| SA0253 | *lrgB* | T,O,D | 2.9, 3.1, 2.8 | SarA- | m (7) | Antiholin-like protein LrgB | H |
| SA0262 |  | T,O,D | 2.4, 2.4, 2.2 |  | c | Hypothetical protein | K |
| SA0271 |  | T,O,D | 2.9, 2.6, 2.4 | Agr+/SarA- | c | Conserved hypothetical protein | K |
| SA0272 |  | T,O | 2.1, 2.5 |  | m (6) | Similar to transmembrane protein Tmp7 | K |
| SA0309 | *geh* | T,O | 2.2, 2.2 | Agr+ | c | Glycerol ester hydrolase | F |
| SA0322 |  | T,O | 4.5, 4.2 |  | c | Similar to transcription regulator | C |
| SA0373 | *xprT* | T,O | 2.3,2.0 |  | c | Xanthine phosphoribosyltransferase | F |
| SA0393 | *ssl11 (set15)* | T,O,D | 9.1, 19.1, 6.2 | VMC+/ SaeRS+ | e | Staphylococcal superantigen-like protein 11 (Exotoxin 15) | J |
| SA0423 |  | T,O,D | 7.9, 17.3, 2.6 | VMC- | m (1) | Similar to autolysin (N-acetylmuramoyl-L-alanine amidase | H |
| SA0456 | *spoVG* | T,O,D | 3.3, 3.7, 3.9 |  | c | Stage V sporulation protein G homologue | K |
| SA0562 | *adh1* | T,O,D | 3.2, 3.4, 3.1 |  | c | Alcohol dehydrogenase | F |
| SA0620 |  | T,O,D | 3.3, 4.3, 2.1 |  | e? | Secretory antigen SsaA homologue | H |
| SA0641 |  | T,O,D | 2.3, 2.0, 2.2 |  | c | Similar to transcriptional regulator | C |
| SA0653 |  | T,O,D | 3.0, 2.4, 2.0 |  | c | Similar to transcription repressor of fructose operon | C |
| SA0654 | *fruB* | T,O,D | 3.0, 2.2, 2.0 |  | c | Fructose 1-phosphate kinase | F |
| SA0655 | *fruA* | T,O,D | 2.7, 2.4, 2.0 |  | m (9) | Fructose specific permease | G |
| SA0660 | *saeS* | T,O,D | 2.3, 2.9, 2.4 | VMC+/  SaeRS+ | m (2) | Histidine protein kinase | B,C |
| SA0661 | *saeR* | T,O,D | 2.0, 2.6, 2.1 | VMC+ | c | Response regulator | B,C |
| SA0663 |  | T,O,D | 3.7, 5.5, 3.5 | SaeRS+ | m (lp)? | Hypothetical protein | K |
| SA0739 |  | T,O,D | 2.9, 3.7, 2.6 |  | c | Hypothetical protein | K |
| SA0742 | *clfA* | T,O,D | 2.2, 2.1, 2.0 |  | e (w) | Fibrinogen-binding protein A, clumping factor | J |
| SA0821 | *argH* | T,O,D | 2.8, 3.7, 6.1 |  | c | Argininosuccinate lyase | E |
| SA0822 | *argG* | T,O,D | 2.5, 3.3, 6.0 |  | c | Argininosuccinate synthase | E |
| SA0890 |  | T,O,D | 2.9, 2.1, 2.8 |  | m (2) | Conserved hypothetical protein | K |
| SA0905 | *atl* | T,O,D | 2.5, 2.5, 2.2 | SarA-/  VMC- | e? | Autolysin | H |
| SA0910 |  | T,O,D | 3.1, 3.4, 2.9 |  | m (3) | Similar to quinol oxidase polypeptide IV QoxD | F |
| SA0911 | *qoxC* | T,O,D | 2.5, 2.8, 2.5 |  | m (5) | Quinol oxidase polypeptide III QoxC | F |
| SA0912 | *qocB* | T,O,D | 2.4, 2.8, 2.4 |  | m (15) | Quinol oxidase polypeptide I QoxB | F |
| SA0913 |  | T,O,D | 2.5, 2.8, 2.4 |  | m (3) | Similar to quinol oxidase polypeptide II QoxA | F |
| SA0916 |  | T,O,D | 3.2, 3.7, 2.1 |  | c | Similar to phosphoribosylaminoimidazole carboxylase PurE | F |
| SA0917 | *purK* | T,O,D | 4.7, 4.6, 2.9 |  | c | Phosphoribosylaminoimidazole carboxylase carbon dioxide-fixation chain PurK homologue | F |
| SA0918 | *purC* | T,O,D | 5.0, 4.9, 3.9 |  | c | Phosphoribosylaminoimidazole-succinocarboxamide synthase | F |
| SA0919 |  | T,O,D | 3.8, 3.3, 3.4 |  | c | Hypothetical protein | K |
| SA0920 | *purQ* | T,O,D | 3.8, 3.9, 3.4 |  | c | Phosphoribosylformylglycinamidine synthase I PurQ | F |
| SA0921 | *purL* | T,O,D | 2.3, 2.6, 2.6 |  | c | Phosphoribosylformylglycinamidine synthetase PurL | F |
| SA0965 | *ctaB* | T,O | 2.0, 2.3 |  | m (8) | Protoheme IX farnesyltransferase | F |
| SA0966 |  | T,O | 2.4, 2.4 |  | m (4) | Conserved hypothetical protein | K |
| SA1016 |  | T,O | 3.3, 3.1 |  | m (10) | Conserved hypothetical protein | K |
| SA1149 | *glnR* | T,D | 3.1, 2.1 |  | c | Glutamine synthetase repressor | C |
| SA1150 | *glnA* | T | 2.5 |  | c | Glutamine-ammonia ligase | E,F |
| SA1168 |  | T,O | 2.4, 2.6 |  | c | Hypothetical protein | K |
| SA1174 | *lexA* | T,O | 2.9, 3.0 |  | c | LexA repressor | C |
| SA1178 |  | T,O,D | 2.5, 2.5, 2.3 |  | m (1)/e? | Conserved hypothetical protein | K |
| SA1234 | *cspA* | T,O,D | 2.9, 2.3, 2.4 |  | c | Major cold shock protein CspA | K |
| SA1251 | *murG* | T,O | 2.0, 2.2 |  | c | N-acetylglucosaminyl transferase | H |
| SA1270 |  | T,O,D | 2.7, 3.6, 2.8 |  | m (12) | Similar to amino acid pearmease | G |
| SA1271 |  | T,O,D | 3.0, 4.0, 3.1 |  | c | Threonine dehydratase | E |
| SA1272 |  | T,O,D | 6.2, 9.8, 7.5 | Agr- | c | Alanine dehydrogenase | E,F |
| SA1275 |  | T,O,D | 3.9, 6.5, 2.4 | Agr+/SarA+ | m (5) | Conserved hypothetical protein | K |
| SA1452 | *csbD* | T,O,D | 2.2, 2.1, 2.0 |  | c | SigmaB-controlled gene product |  |
| SA1493 | *hemD* | T,O | 2.5, 2.9 |  | c | Uroporphyrinogen III synthase | F |
| SA1494 | *hemC* | T,O,D | 2.3, 2.4, 2.1 |  | c | Porphobilinogen deaminase | F |
| SA1495 | *hemX* | T,O | 2.1, 2.4 |  | m (8) | HemA concentration negative effector HemX | C |
| SA1496 | *hemA* | T,O | 2.4, 2.4 |  | c | Glutamyl-tRNA reductase | F |
| SA1665 |  | T,O | 2.4, 2.2 | VMC- | c | Hypothetical protein | K |
| SA1674 |  | T,O,D | 3.4, 3.5, 4.0 |  | c | Glutamate ABC transporter ATP-binding protein | G |
| SA1675 |  | T,O,D | 3.9, 3.6, 5.9 |  | m (3) | Similar to glutamine-binding periplasmic protein | G |
| SA1730 |  | T,O,D | 2.4, 2.1, 2.0 |  | c | Nitric-oxide synthase homologue | F |
| SA1751 | *mapW* | T,O,D | 2.2, 2.7, 2.3 | SaeRS+ | e | Truncated map-w protein | K |
| SA1754 |  | T,O,D | 5.5, 5.5, 4.0 |  | c | Hypothetical protein | K |
| SA1810 | *int* | T,O | 2.1, 2.8 |  | c | Integrase | C |
| SA1812 |  | T,O,D | 2.7, 4.7, 2.6 |  | e | Similar to synergohymenotropic toxin precursor - *Staphylococcus intermedius* | J |
| SA1813 |  | T,O,D | 3.4, 5.3, 3.2 |  | e | Similar to leukocidin chain lukM precursor | J |
| SA1843 | *agrC* | T,O,D | 3.9, 6.1, 3.7 | Agr+/SarA+ | m (4) | Accessory gene regulator C | B,C |
| SA1844 | *agrA* | T,O,D | 3.1, 3.8, 2.6 | Agr+/SarA+/SaeRS+ | c | Accessory gene regulator A | B,C |
| SA1849 |  | T,O | 2.0, 2.1 |  | c | Hypothetical protein | K |
| SA1975 |  | T,O | 2.5, 2.1 |  | m (4) | Conserved hypothetical protein | K |
| SA1959 | *glmS* | T,O,D | 2.6, 2.2, 2.1 |  | c | glucosamine-fructose-6-phosphate aminotransferase | F |
| SA2007 |  | T,O,D | 3.0, 2.8, 2.3 |  | c | Similar to alpha-acetolactate decarboxylase | F |
| SA2008 | *alsS* | T,O | 3.4, 2.1 |  | c | Alpha-acetolactate synthase | F |
| SA2050 |  | T,O,D | 2.7, 2.3, 2.0 |  | m (13) | Conserved hypothetical protein | G |
| SA2093 | *ssaA* | T,O,D | 4.9, 10.3, 2.2 | Agr-/  VMC- | e | Secretory antigen precursor SsaA homologue | J |
| SA2097 |  | T,O,D | 2.7, 2.4, 2.2 |  | c/e? | Similar to secretory antigen precursor SsaA | K |
| SA2108 |  | T,O,D | 2.9, 2.6, 2.1 |  | c | Similar to transcription regulator, RpiR family | C |
| SA2126 |  | T,O,D | 2.9, 2.5, 2.0 | VMC- | m (8) | Hypothetical protein | K |
| SA2156 |  | T,O,D | 8.6, 4.0, 6.4 |  | m (13) | L-lactate permease lctP homologue | G |
| SA2163 |  | T,O,D | 2.4, 2.3, 2.0 |  | m (4) | Hypothetical protein | K |
| SA2176 | *narK* | T,O,D | 6.1, 6.0, 3.8 |  | m (12) | Nitrite extrusion protein | G |
| SA2179 |  | T,O,D | 4.6, 5.4, 3.4 |  | c | Similar to response regulators of two-component regulatory | B,C |
| SA2180 |  | T,O,D | 3.0, 4.4, 2.0 |  | c? | Similar to two component sensor histidine kinase | B,C |
| SA2181 |  | T,O,D | 2.6, 3.6, 2.1 |  | c | Hypothetical protein | K |
| SA2182 | *narI* | T,O,D | 4.2, 7.1, 2.7 |  | m (5) | Nitrate reductase gamma chain | F |
| SA2183 |  | T,O,D | 5.8, 22.9, 3.6 |  | c | Similar to nitrate reductase delta chain | F |
| SA2184 | *narH* | T,O,D | 5.6, 20.5, 3.2 |  | c | Nitrate reductase beta chain narH | F |
| SA2185 | *narG* | T,O,D | 5.4, 12.3, 3.0 | Agr- | c | Respiratory nitrate reductase alpha chain | F |
| SA2186 | *nasF* | T.O,D | 13.1, 17.4, 5.8 |  | c | Uroporphyrin-III C-methyl transferase | F |
| SA2187 | *nasE* | T,O,D | 8.7, 10.2, 4.1 | Agr- | c | Assimilatory nitrite reductase | F |
| SA2188 | *nasD* | T,O,D | 17.8, 16.0, 5.4 |  | c | Nitrite reductase | F |
| SA2189 |  | T,O,D | 21.9, 13.5, 6.0 |  | c | Similar to NirR | C |
| SA2197 |  | T,O | 3.0, 2.5 |  | m (lp)? | Conserved hypothetical protein/putative protein-disulfide isomerase | A |
| SA2198 |  | T,O | 2.4, 2.3 |  | m (lp)? | Hypothetical protein | K |
| SA2206 | *sbi* | T,O,D, | 3.8, 5.9, 2.8 | VMC+/  SaeRS+ | e? | IgG-binding protein SBI | J |
| SA2207 | *hlgA* | T,O,D | 5.5, 6.6, 5.8 | VMC+/  SaeRS+ | e | Gamma-hemolysin chain II precursor | J |
| SA2221 |  | T,O | 3.0, 2.3 |  | m (1) | Hypothetical protein | K |
| SA2246 |  | T,O | 2.0, 2.2 |  | c | Hypothetical protein | K |
| SA2268 |  | T,O,D | 5.3, 5.6, 4.2 |  | c | Hypothetical protein | K |
| SA2302 |  | T,O,D | 2.2, 3.1, 2.2 |  | c | Similar to ABC transporter | G |
| SA2303 |  | T,O,D | 2.0, 2.3, 2.2 |  | m (6) | Similar to membrane spanning protein | G |
| SA2317 |  | T,O | 2.7, 2.3 |  | c | Similar to N-acetyltransferase | K |
| SA2326 | *ptsG* | T,O,D | 2.2, 2.3, 2.2 |  | m (9) | PTS system, glucose-specific IIABC component | F,G |
| SA2336 | *clpL* | T,O,D | 3.2, 6.7, 6.0 | SarA- | c | ATP-dependent Clp proteinase chain clpL | D |
| SA2338 |  | T,O | 4.2, 2.8 |  | c | Hypothetical protein/FeoA domain | K |
| SA2340 |  | T,O,D | 4.1, 3.2, 2.0 |  | c | Similar to transcriptional regulator tetR-family | C |
| SA2355 |  | T,O | 4.5, 7.7 | VMC- | c | Hypothetical protein/similar to acetyltransferase | K |
| SA2356 | *isaA* | T,O | 2.7, 3.6 | VMC- | e? | Immunodominant antigen A | K |
| SA2423 | *clfB* | T,O | 2.1, 2.5 |  | e (w) | Clumping factor B | J |
| SA2425 | *arcC* | T | 2.5 | Agr+/SarA- |  | Carbamate kinase | E,F |
| SA2426 | *arcD* | T,O,D | 2.8, 2.4, 3.0 | Agr+ | m (14) | Arginine/oirnithine antiporter | G |
| SA2427 | *arcB* | T,D | 2.0, 2.0 | Agr+ | c | Ornithine carbamoyltransferase | E |
| SA2447 |  | T,O | 2.1, 2.4 | Agr+/SarA+  VMC- | c (w) | Similar to streptococcal hemagglutinin protein | J |
| SA2494 | *cspB* | T,D | 2.2, 2.1 |  | c | Cold shock protein cspB | K |
| SA2496 |  | T,O,D | 2.3, 2.1, 2.2 |  | m (4) | Hypothetical proteins | K |
| SAS009 |  | T,O | 2.8, 2.3 |  | c | Hypothetical protein | K |
| SAS041 |  | T,O,D | 5.1, 5.3, 4.1 |  | m (2) | Hypothetical protein | K |
| SAS049 |  | T,O,D | 2.5, 2.4, 2.2 |  | c | Hypothetical protein | K |
| SAS065 | *hld* | T,O,D | 8.4, 24.7, 5.8 | Agr+/SarA+ | c | Delta-hemolysin | J |
| SAS068 |  | T,O,D | 3.1, 3.2, 2.1 |  | c | Hypothetical protein | K |
| *: T, temporin L-NH_2_; O, ovispirin-1-NH_2_; D, dermaseptin K4-S4(1-16)-NH_2_;  **†:** Induced (+) or repressed (-) by vancomycin (VCM) or regulator;  §: m, predicted membrane protein, number of predicted transmembrane segments is in parenthesis; c, predicted cytoplasmic protein; e, exported protein; lp, lipoprotein; w, wall-asociated protein;  ¶: FC, Functional category; A, Stress combating mechanisms, protein folding, chaperon function; B, Signal transduction (two-component systems); C, Transcription, transcription regulation, replication, integration, nucleic acid metabolism, translation, ribosomes; D, Proteolysis, peptide cleavage; E, Amino acid metabolism and biogenesis; F, Other metabolic/biosynthetic functions (e.g. energy metabolism and carbohydrate metabolism); G, Molecule/ion uptake, export, protein secretion; H, Cell wall structure, biogenesis, hydrolysis and modification, cell division; I, Protein modification; J, Virulence and pathogenesis; K, Functionally unknown proteins, hypothetical proteins with conserved domains, hypothetical proteins. | | | | | | | |
