# Supplementary material for: Transcriptome analysis of the responses of Staphylococcus aureus to antimicrobial peptides and characterization of the roles of vraDE and vraSR in antimicrobial resistance
Source: BMC Genomics. 2009 Sep 14;10:429. doi: 10.1186/1471-2164-10-429 (PMC2748101; doi:10.1186/1471-2164-10-429)
Supplement: Additional file 3 — Antimicrobial sensitivity of S. aureus RN4220 and its ΔvraDE and ΔvraSR mutant derivatives. The table shows MIC values of antimicrobial peptides and other antimicrobial agents against S. aureus RN4220 and its ΔvraDE and ΔvraSR mutant derivatives. [file 1471-2164-10-429-S3.doc]

| **Table S3: Antimicrobial sensitivity of *S. aureus* RN4220 and its Δ*vraDE* and Δ*vraSR* mutant derivatives.** | | | |
| --- | --- | --- | --- |
| **Antimicrobial agent** | **MIC (μg ml^-1^)** | | |
|  | Δ*vraSR*/  RN4220 | Δ*vraDE*/  RN4220 | *S. aureus* RN4220 |
| Ovispirin-1 | 20 | 20 | 20 |
| Ovispirin-1-NH_2_ | 10 | 20 | 20 |
| Temporin L | 3 | 3 | 3 |
| Nisin | 6.25 | 6.25 | 6.25 |
| Bacitracin | 5 | 2.5 | 20 |
| Daptomycin | 2.5 | 2.5 | 2.5 |
| Teicoplanin | 0.06 | 0.25 | 0.25 |
| Vancomycin | 0.5 | 1 | 1 |
