# Supplementary material for: Transcriptome analysis of the responses of Staphylococcus aureus to antimicrobial peptides and characterization of the roles of vraDE and vraSR in antimicrobial resistance
Source: BMC Genomics. 2009 Sep 14;10:429. doi: 10.1186/1471-2164-10-429 (PMC2748101; doi:10.1186/1471-2164-10-429)

**Additional file 4:** Antimicrobial sensitivities of RH7657 (*vraSR::ery*) and RH7603 (*S. aureus* Newman) were compared by using phenotype microarrays. Scatter plots of parameter values from two replicates of the PM analysis are shown in the two uppermost panels. The three other panels show overlaid color-coded images of tetrazolium reduction kinetics (mutant versus wild type) over all wells in the two runs of the analysis and their consensus.

Replicate 1 versus Replicate 2 of RH7657 (*vraSR::ery*)


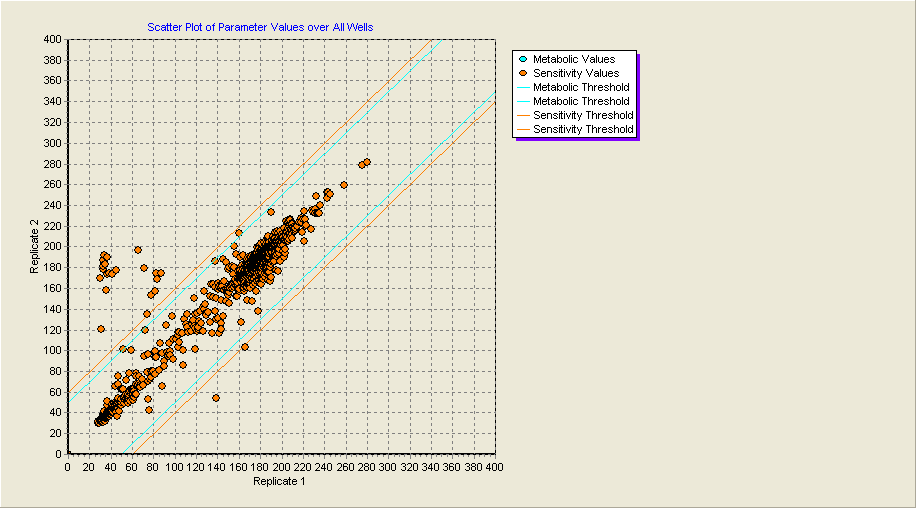


Replicate 1 versus Replicate 2 of RH7603 (*S. aureus* Newman)


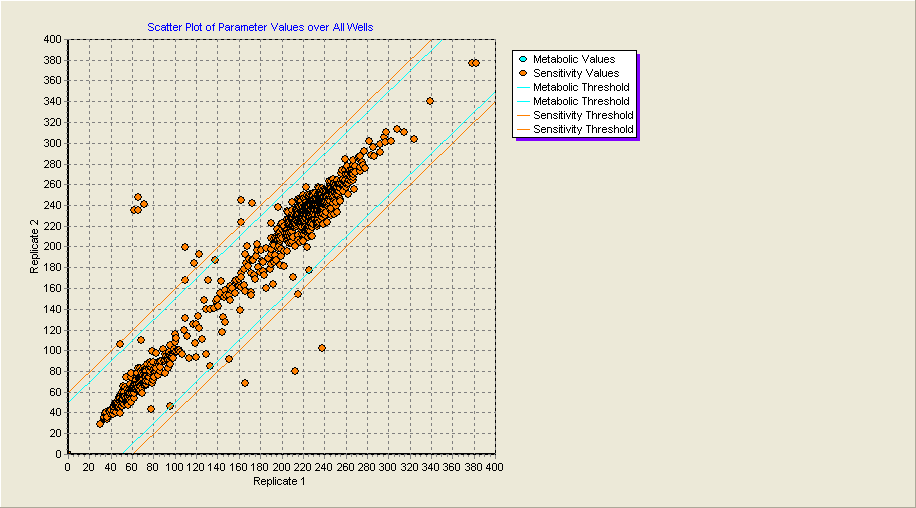


Run 1: RH7657 (green) versus RH7603 (red)


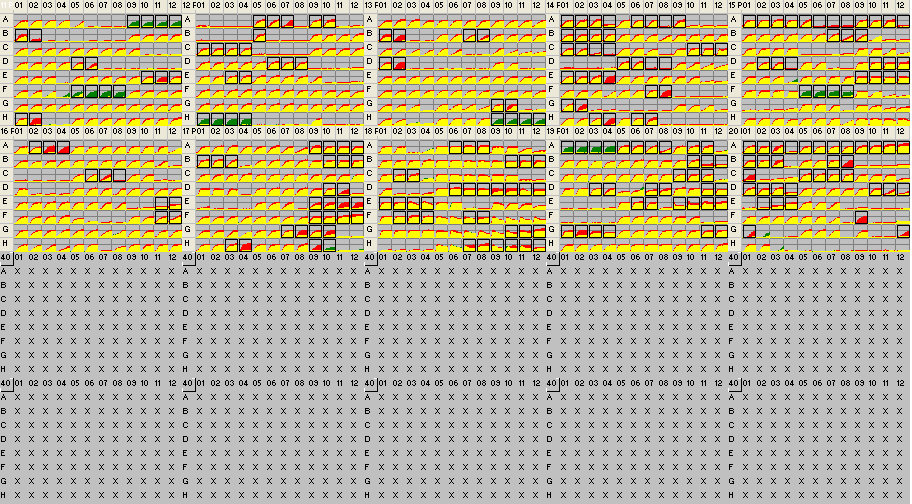


Run 2: RH7657 (green) versus RH7603 (red)


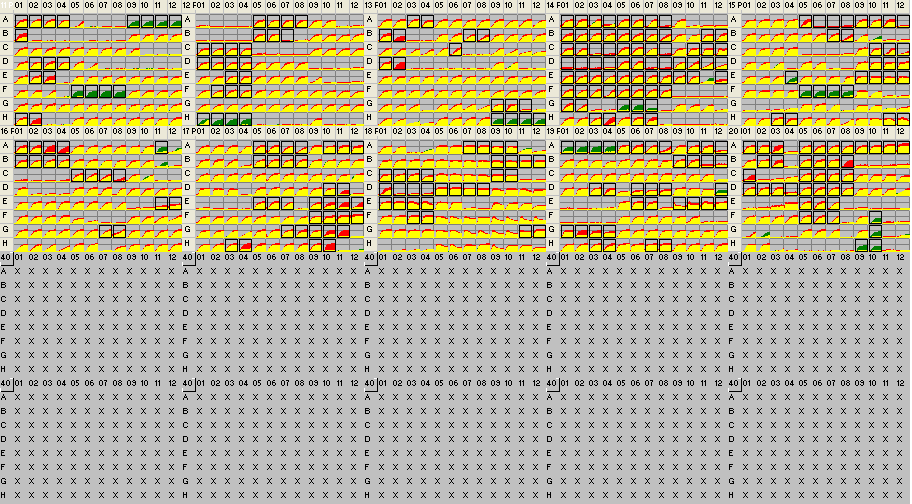


Consensus: RH7657 (green) versus RH7603 (red)


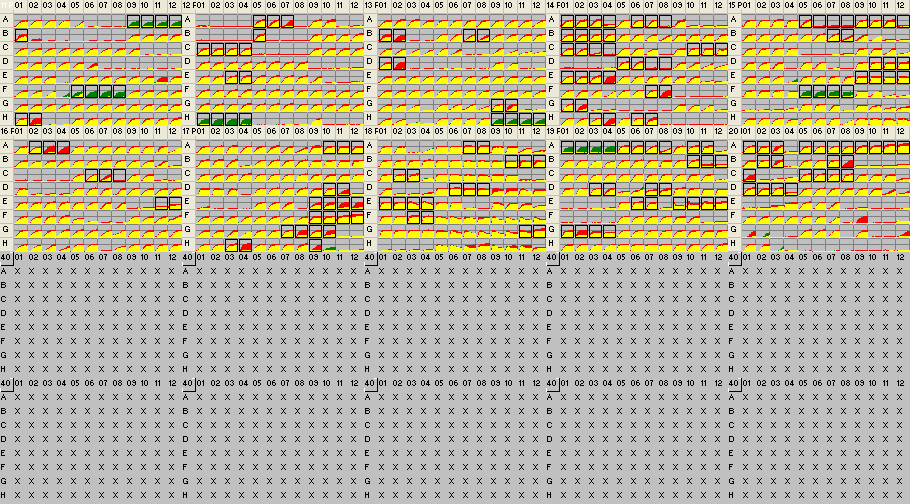

Supplement: Additional file 4 — Antimicrobial sensitivities of RH7657 (vraSR::ery) and RH7603 (S. aureus Newman) were compared by using phenotype microarrays. Scatter plots of parameter values from two replicates of the PM analysis are shown in the two uppermost panels. The three other panels show overlaid color-coded images of tetrazolium reduction kinetics (mutant versus wild type) over all wells in the two runs of the analysis and their consensus. [file 1471-2164-10-429-S4.doc]
