# Supplementary material for: Transcriptome analysis of the responses of Staphylococcus aureus to antimicrobial peptides and characterization of the roles of vraDE and vraSR in antimicrobial resistance
Source: BMC Genomics. 2009 Sep 14;10:429. doi: 10.1186/1471-2164-10-429 (PMC2748101; doi:10.1186/1471-2164-10-429)
Supplement: Additional file 5 — Decreased reduction of tetrazolium in vraSR null mutant treated with antimicrobial agents as compared to the wild-type S. aureus Newman strain. The gene list was obtained from the consensus of the two independent phenotype microarray analyses shown in additional file 4. [file 1471-2164-10-429-S5.doc]

**Additional file 5:** Decreased reduction of tetrazolium in *vraSR* null mutant treated with antimicrobial agents as compared to the wild-type *S. aureus* Newman strain. The list of the antimicrobial agents was obtained from the consensus of the two independent phenotype microarray analyses shown in additional file 4.

| **Plate** | **Wells** | **Antimicrobial agent** | **Functional role/target/inhibitor of** |
| --- | --- | --- | --- |
| PM12B | E03,E04 | 2,4-Diamino-6,7-diisopropylpteridine |  |
| PM20B | C05,C06,C07,C08 | Atropine | acetylcholine receptor, antagonist |
| PM17A | A10,A11,A12 | Thiosalicylate | anti-capsule, thiol |
| PM20B | A05,A06,A07,A08 | Apramycin | antimicrobial, aminocyclitol |
| PM17A | F09,F10,F11,F12 | Tannic acid | antimicrobial, from plants |
| PM19 | A05,A06,A07,A08 | Gallic acid | antimicrobial, from plants |
| PM20B | B07,B08 | D,L-Propranolol | beta-adrenergic blocker |
| PM18C | F03,F04 | Semicarbazide hydrochloride | carbonyl agent, semicarbazide sensitive amine oxidase, DNA damage |
| PM13B | G09,G10 | Trifluoperazine | cell cycle modulation, DNA synthesis, Ca(2+)/calmodulin dependent protein phosphorylation |
| PM18C | A07,A08 | Sodium pyrophosphate decahydrate | chelating agent |
| PM14A | H03,H04 | EGTA | chelator, Ca++ |
| PM13B | B07,B08 | 2,2`-Dipyridyl | chelator, Fe++ |
| PM15B | B07,B08 | EDTA | chelator, hydrophilic |
| PM20B | B02,B03 | Orphenadrine | cholinergic antagonist |
| PM14A | H06,H07 | Promethazine | cyclic nucleotide phosphodiesterase |
| PM18C | G11,G12 | Myricetin | DNA & RNA synthesis, polymerase inhibitor (*E. coli*) |
| PM14A | A01,A02,A03,A04 | Acriflavine | DNA intercalator |
| PM14A | B01,B02,B03,B04 | 9-Aminoacridine | DNA intercalator |
| PM19 | A10,A11 | Coumarin | DNA intercalator |
| PM14A | A06,A07,A08 | Furaltadone | DNA synthesis, nitro-compound, multiple sites |
| PM20B | A09,A10,A11,A12 | Benserazide | fungicide |
| PM19 | D03,D04 | Disulphiram | fungicide |
| PM15B | D10,D11,D12 | Nordihydroguaiaretic acid | lipoxygenase, fungicide |
| PM16A | C06,C07,C08 | Protamine sulfate | membrane, ATPase |
| PM15B | A05,A06,A07,A08 | Guanidine hydrochloride | membrane, chaotropic agent |
| PM18C | C02,C03 | Poly-L-lysine | membrane, detergent, cationic |
| PM19 | G01,G02,G03,G04 | Lauryl sulfobetaine | membrane, detergent, zwitterionic |
| PM20B | A01,A02,A03 | Amitriptyline | membrane, transport |
| PM18C | B10,B11,B12 | Azathioprine | nucleic acid analog, purine |
| PM14A | D05,D06,D07,D08 | Iodoacetate | oxidation, sulfhydryl |
| PM19 | E09,E10,E11,E12 | Lawsone | oxidizing agent |
| PM15B | E09,E10,E11,E12 | Methyl viologen | oxidizing agent |
| PM19 | E06,E07 | D,L-Thioctic acid | oxidizing agent |
| PM17A | D10,D11 | Chlorpromazine | phenothiazine |
| PM14A | G01,G02 | Chelerythrine | protein kinase C |
| PM12B | C01,C02,C03,C04 | Paromomycin | protein synthesis, aminoglycoside |
| PM12B | A05,A06,A07 | Tetracycline | protein synthesis, tetracycline |
| PM12B | B05 | Penimepicycline | protein synthesis, tetracycline |
| PM20B | C01 | Thioridazine | respiration |
| PM19 | D07,D08 | Iodonitro tetrazolium violet | respiration |
| PM19 | B10,B11,B12 | 2,4-Dintrophenol | respiration, ionophore, H+ |
| PM16A | E11,E12 | Rifamycin SV | RNA polymerase |
| PM20B | D01,D02,D03,D04 | Proflavine | RNA synthesis |
| PM18C | E01,E02,E03,E04 | Sodium metasilicate | toxic anion |
| PM18C | D06,D07,D08 | Sodium bromate | toxic anion |
| PM14A | C01,C02,C03,C04 | Boric acid | transport, toxic anion |
| PM14A | C10,C11,C12 | Sodium cyanate | transport, toxic anion |
| PM17A | E09,E10,E11,E12 | Sodium tungstate | transport, toxic anion, molybdate analog |
| PM17A | H09 | Phenylarsine oxide | tyrosine phosphatase |
| PM16A | A02,A03,A04 | Cefotaxime | wall, cephalosporin |
| PM14A | E01,E02,E03,E04 | Cefoxitin | wall, cephalosporin |
| PM17A | H03,H04 | Cefsulodin | wall, cephalosporin |
| PM17A | G07,G08 | Cefamandole | wall, cephalosporin |
| PM17A | G09,G10 | Cetoperazone | wall, cephalosporin |
| PM15B | A09,A10,A11,A12 | Cefmetazole | wall, cephalosporin second generation |
| PM13B | D01,D02 | Cefuroxime | wall, cephalosporin second generation |
| PM13B | B01,B02 | Azlocillin | wall, lactam |
| PM14A | F07,F08 | Piperacillin | wall, lactam |
| PM11C | H01,H02 | Cephalothin | wall, cephalosporin first generation |
| PM11C | B01 | Amoxicillin | wall, lactam |
