# Supplementary material for: Transcriptome analysis of the responses of Staphylococcus aureus to antimicrobial peptides and characterization of the roles of vraDE and vraSR in antimicrobial resistance
Source: BMC Genomics. 2009 Sep 14;10:429. doi: 10.1186/1471-2164-10-429 (PMC2748101; doi:10.1186/1471-2164-10-429)
Supplement: Additional file 6 — Specific primer pairs for qRT-PCR and PCR. The table shows the primers used in this study. [file 1471-2164-10-429-S6.doc]

| **Table S4: Specific primer pairs for QRT-PCR and PCR.** | | |
| --- | --- | --- |
| **Target gene** | **Forward primer** | **Reverse primer** |
| **Primers for quantitative real-time RT-PCR** | |  |
| *gyrA* | 5’-ggacgtcaacgtattgttgtcact-3’ | 5’- cgagctctgcaattttttcaatc-3’ |
| SA0205 | 5’-gcatattcaggcaatacaggtaaaca-3’ | 5’-cattacctacgccacccttca-3’ |
| SA0423 | 5’-caagcaaatgcggctacaac-3’ | 5’-gaaattgcccacactgattcac-3’ |
| SA0616 | 5’-gcatgatcctgttgcagcaa-3’ | 5’-tccccttggtataattcagtgaaaa-3’ |
| SA0793 | 5’-agcgttagtgagccgtttcc-3’ | 5’-cgtagcttcagttggaccatatgt-3’ |
| SA1220 | 5’-tcgttctcgcgatttcaaga-3’ | 5’-aactacctgccgctaatgatacaa-3’ |
| SA1477 | 5’-gagttggactttctattttgcgatt-3’ | 5’-cgagcaaccaaattaaaccttga-3’ |
| SA1659 | 5’-gacagcaagaaagcttcacacatt-3’ | 5’-tgaatttcttcagctttttgtttcg-3’ |
| SA1701 | 5’-tgcgtgaaagagctttggaa-3’ | 5’-gtacctgaatctggcaatgatacaa-3’ |
| SA1836 | 5’-gaaaatgcaggattagaaggttctg-3’ | 5’-tgtagcagcgttaaaaccaacac-3’ |
| SA2093 | 5’-ggctaacgcagctgcaagag-3’ | 5’-tttgcataattgcaccagcttt-3’ |
| SA2206 | 5’-atacatcaaaacattacgcgaacac-3’ | 5’-gggttcttgctgtctttaagtgatt-3’ |
| SA2492 | 5’-tgcagcagcgagagcattt-3’ | 5’-cctgttggctcatctgcaaa-3’ |
| SAS016 | 5’-gattatttatcgacagtatcaccatga-3’ | 5’-ttaattgaaacatgctgaaacgtt-3’ |
| **Primers for PCR** | | |
| erythromycin resistance gene from pMUTIN4 | 5’-cacgtcgaccacctgacgtctaagaaacc-3’ | 5’-cacggatcctctagagcaacgttcttgcc-3’ |
| ***vraDE* deletion:** | | |
| flanking area upstream from *vraDE* | 5’-cacactgcagacaatcatttggccgtgctac-3’ | 5’-cacagtcgaccactgataatatcgtcatagtct  cactcc-3’ |
| flanking area downstream from *vraDE* | 5’-cacaggatccacaggcaacaatgccatcattac-3’ | 5’-cacagaattccgccacctaaggaatgcc-3’ |
| *vraDE* deletion cassette for pKOR1 | 5’-gggacaagtttgtacaaaaaagcaggct  acaatcatttggccgtgctac-3’ | 5’-ggggaccactttgtacaagaaagctgggt  cgccacctaaggaatgcc-3’ |
| verification of the Δ*vraDE* mutation | 5’-caattactgatcattccgcgcta-3’  5’-acatttcgaacctatggccc-3’ | 5’-gcactaccatgacgacgacc-3’ |
| ***vraSR* deletion:** | | |
| flanking area upstream from *vraSR* | 5’-caacaaggatccctaagatgagcattgaacc-3’ | 5’-caacaagagctcgaacacaatcaatgcgtc-3’ |
| flanking area downstream from *vraSR* | 5’-caacaactgcagcaatgttgcttacgatgtag-3’ | 5’-caacaagtcgaccttacagaacgagaaatgg-3’ |
| *vraSR* deletion cassette for pKOR1 | 5’-gggacaagtttgtacaaaaaagcaggct  caatgttgcttacgatgtac-3’ | 5’-ggggaccactttgtacaagaaagctgggt  gaacacaatcaatgcgtc-3’ |
| verification of the Δ*vraSR* mutation | 5’-cttcaaatggaatgactgc-3’  5’-cagaatcgtgaagttctcgagc-3’ | 5’-ggagcctattcatattgg-3’ |
